# Proteomic biomarkers of long-term lung function decline in textile workers: a 35-year longitudinal study

**DOI:** 10.1038/s41370-024-00721-7

**Published:** 2024-10-02

**Authors:** Mengsheng Zhao, Liangmin Wei, Longyao Zhang, Jingqing Hang, Fengying Zhang, Li Su, Hantao Wang, Ruyang Zhang, Feng Chen, David C. Christiani, Yongyue Wei

**Affiliations:** 1 Department of Biostatistics, Center for Global Health, School of Public Health, Nanjing Medical University, Nanjing, China; 2 Department of Public Health, School of Medicine & Holistic Integrative Medicine, Nanjing University of Chinese Medicine, Nanjing, China; 3 Department of Pulmonary Medicine, Shanghai Putuo District People’s Hospital, Shanghai, China; 4 Department of Environmental Health, Harvard T.H. Chan School of Public Health, Boston, MA USA; 5 Pulmonary and Critical Care Division, Department of Medicine, Massachusetts General Hospital and Harvard Medical School, Boston, MA USA; 6 Center for Public Health and Epidemic Preparedness & Response, Peking University, Beijing, China; 7 Key Laboratory of Epidemiology of Major Diseases (Peking University), Ministry of Education, Beijing, China

**Keywords:** Proteomics, Biomarkers, Lung function, Textile workers

## Abstract

**Background:**

Occupational exposures contribute significantly to obstructive lung disease among textile workers. However, biomarkers associated with such declines are not available.

**Objectives:**

We conducted a large-scale proteomic study to explore protein biomarkers potentially associated with long-term lung function decline.

**Methods:**

Shanghai Textile Workers Cohort was established in 1981 with 35 years of follow-up, assessing textile workers’ lung functions every five years. Quantitative serum proteomics was performed on all 453 workers at 2016 survey. We employed four distinct models to examine the association between forced expiratory volume in one second (FEV_1_) and proteins, and consolidated the findings using an aggregated Cauchy association test. Furthermore, proteomic data of UK Biobank (UKB) was used to explore the associations of potential protein markers and decline of FEV_1_, and the interactions of these proteins were examined through STRING database. Associations were also externally validated using two-sample Mendelian randomizations (MR).

**Results:**

15 of 907 analyzed proteins displayed potential associations with long-term FEV_1_ decline, including two hemoglobin subunits: hemoglobin subunit beta (HBB, FDR-*q*_ACAT_ = 0.040), alpha globin chain (HBA2, FDR-*q*_ACAT_ = 0.045), and four immunoglobulin subunits: immunoglobulin kappa variable 3–7 (IGKV3-7, FDR-*q*_ACAT_ = 0.003), immunoglobulin heavy chain variable region (IgH, FDR-*q*_ACAT_ = 0.011). Five proteins were significantly associated with the rate of decline of FEV_1_ in UKB, in which RAB6A, LRRN1, and BSG were also found to be associated with proteins identified in Shanghai Textile Workers Cohort using STRING database. MR indicated bidirectional associations between HBB and FEV_1_ (*P* < 0.05), while different immunoglobulin subunits exhibited varying associations with FEV_1_.

**Impact statement:**

We performed a large-scale proteomic study of the longest-follow-up pulmonary function cohort of textile workers to date. We discovered multiple novel proteins associated with long-term decline of FEV_1_ that have potential for identifying new biomarkers associated with long-term lung function decline among occupational populations, and may identify individuals at risk, as well as potential pharmaceutical targets for early intervention.

## Introduction

Chronic obstructive pulmonary disease (COPD) is now the third leading cause of death worldwide, accounting for 3.3 million fatalities in 2019 [1]. While cigarette smoking has conventionally been regarded as the preeminent risk factor of COPD, causative factors other than tobacco smoking have been increasingly acknowledged, given that one-third of global COPD patients are non-smokers [2, 3]. Strong evidence from Global Burden of Disease Study 2019 indicates that pollution from ambient particulate matter and occupational exposure to particulate matter, gases, and fumes are also important risk factors contributing to the development of COPD [4].

Occupational exposure to cotton dust can lead to both acute respiratory responses [5] and chronic respiratory diseases [6]. In the early stage of exposure, the symptoms tend to manifest as reversible airflow obstruction [7], while in later stage, symptoms resemble COPD, characterized by fixed airflow obstruction and, notably, an accelerated decline in forced expiratory volume in one second (FEV_1_) [8]. Both experimental and observational studies provide evidence that it is the gram-negative bacterial endotoxin contaminating the cotton dust rather than the dust particle themselves that determines both acute and chronic decline in FEV_1_ [9, 10]. Previous analysis of this study’s cohort, the Shanghai Textile Worker Study, has demonstrated an association between exposure to cotton dust and endotoxin with a decrease of 10 mL/year in 5-year annualized FEV_1_ decline [6], while retirement of textile work was linked to improvements in lung function and respiratory symptoms [11].

Exploring potential biomarkers of FEV_1_ decline may yield new pathobiological insights and identify individuals at risk and, potentially, novel pharmacological targets for early intervention [12]. While several studies have examined specific protein biomarker panels in relation to lung function [13–15], the recent development of large-scale proteomic technologies now enables the concurrent measurement of hundreds to thousands of proteins in epidemiologic cohorts. A recent study on systemic protein biomarkers across six diverse cohorts identified and validated 15 proteins associated with FEV_1_ decline in longitudinal analyses, including elafin leukocyte elastase inhibitor and mucin-associated TFF2 [16]. However, there is limited research available that describes the underlying mechanisms responsible for the decline of FEV_1_ among occupationally exposed populations.

The Shanghai Textile Worker Study is the longest active longitudinal study of textile workers, initiated in 1981 and followed for over 35 years. Remarkably, within this cohort, there have been eight assessments of workers’ lung function over a span of 35 years, with low loss to follow-up. In this study, we aimed to specify novel protein biomarkers associated with FEV_1_ decline within the Shanghai Textile Worker cohort. Furthermore, we conducted Mendelian randomization (MR) analyses in external populations to validate our findings. This study provided mechanistic insights of the decline of FEV_1_ in occupational exposed populations, and provide support for the need to identify individuals at risk, as well as potential pharmaceutical targets for early intervention.

## Methods

Detailed information on participant selection, methods for pulmonary function test, and endotoxin assessment has been described elsewhere [17–19], and are summarized below.

### Study population and study design

**Fig. 1:**
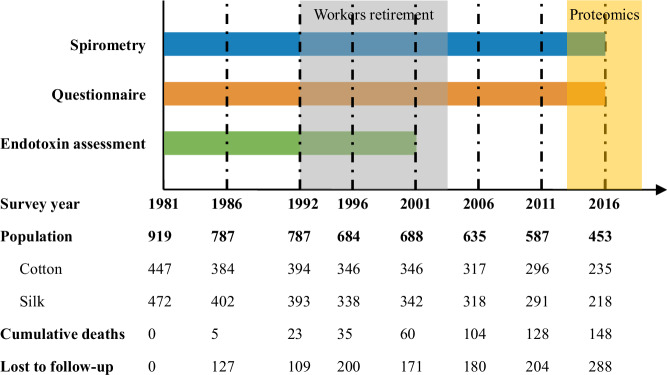
Shanghai textile worker study schema (1981–2016). Most of the workers retired between 1992 and 2001. Serum samples for proteomic analysis were collected from all 453 workers who participated in the 2016 survey.

The Shanghai Textile Worker Study was initiated in 1981. This study included 447 cotton workers who were exposed to airborne cotton dust and endotoxin from two cotton textile mills, alongside 472 unexposed silk workers who worked at a neighboring silk textile mill in Shanghai, China. Participants had no symptoms of respiratory disease and had worked at least 2 years in the industry before the baseline survey. Subsequently, a total of seven follow-up surveys were conducted respectively in 1986, 1992, 1996, 2001, 2006, 2011, and 2016 (see study schema in Fig. 1). Notably, the loss-to-follow-up rate (excluding deaths) still remained around 30% in the last survey at 2016.

### Spirometry measurements

Forced expiratory spirograms were performed both before and after work shifts on the initial workday following a 2-day rest period. Furthermore, all eligible retirees participated in the follow-up cohort surveys. Workers were instructed to abstain from smoking for at least an hour prior to the test. Each worker performed a maximum of seven trials to generate three acceptable curves. The study collected multi-dimensional spirometric metrics like FEV_1_, FVC (forced vital capacity), etc., but primarily focused on indices of FEV_1_. Acceptable FEV_1_ tracings exhibited variations of no more than 5% or 100 mL, whichever was greater. The highest FEV_1_ values from technically acceptable tests were employed in the subsequent analyses.

### Respiratory questionnaire

We used a modified version of the American Thoracic Society (ATS) standardized respiratory symptom questionnaire [20], which was translated into Chinese and subsequently back-translated into English. This questionnaire served as a tool to gather information regarding work, medical, and smoking history at all follow-up surveys, including basic characteristics, working status, retirement date, smoking status, pack-years of smoking, respiratory symptoms, including chronic bronchitis, chronic cough, and dyspnea, and respiratory syndromes, including byssinosis.

### Endotoxin assessment

Airborne cotton dust levels were assessed in the workplace using a Vertical Elutriator during the first four surveys, and the concentrations of gram-negative bacterial endotoxins in the dust samples were determined through the chromogenic assay. In 1996, synthetic fibers were introduced as blends in the cotton mills, resulting in a 50% reduction in both cotton dust and endotoxin exposures compared to pre-blend levels [21]. The airborne endotoxin concentration, estimated from samples collected in the silk mills, closely matched ambient levels. Consequently, silk workers were deemed unexposed to endotoxin.

### Sample collection and proteomics profiling

Peripheral blood samples were collected for every participant when he/she attended the 2016 survey. Subsequently, serum and buffy coat separated from whole blood were stored at −80 °C. Proteins quantification was achieved using data-independent acquisition mass spectrometry, which is a high-throughput proteomics strategy that could accurately quantify proteins with high reproducibility in a complex proteome [22, 23]. Details of the proteomics profiling procedure can be found in supplementary methods.

### Quality control

Protein relative quantitative values were determined by sample normalization and log_2_ transformation. Initially, we excluded proteins lacking annotation data in the UniProt database, as their existence and functional attributes remained uncertain. The proteomic data contained missing values, which could be attributed to the low abundance in certain samples or technical issues. In such instances, we then removed the protein sequences with missing values over 50%. This strategy aimed to exclude protein sequences that were identifiable in only a small number of samples, as they might produce false-positive signals due to technical issues. Subsequently, we considered the complexity of the sources of missing values such as peptide misidentification, below detection limit, incomplete trypsin digestion, etc. Notably, some of these factors are intensity-based, while others are not. Thus, we employed the sequential k-nearest neighbor method for imputing the missing values. Besides, in order to fully consider missingness not at random caused by falling below detection limit, we imputed the missing values with the minimum value identified within the corresponding protein sequences in sensitivity analysis.

### Exploratory study

UK Biobank (UKB) was used as an independent explorative cohort. Within this prospective study, four spirometry tests were conducted among 502,309 individuals, of whom 48,544 had more than two FEV_1_ measurements. We first excluded any participants without at least two spirograms with acceptable starts. For each participant, we then compared each FEV_1_ to their maximum FEV_1_, and spirograms were considered reproducible if they were within 250 mL of the maximum FEV_1_, based on standard spirometry guidelines [24]. Proteomic analysis was conducted on a randomized subset of UKB participants using plasma samples collected during the baseline recruitment phase, involving a total of 53,026 individuals. Proteomic profiling on blood plasma was performed with the Olink Explore 3072 platform, which measures 2923 unique proteins [25]. The protein quantification values obtained were normalized protein expressions. This study included samples of 6177 individuals who had both two or more FEV_1_ measurements and available proteomic data.

### Statistical analysis

**Fig. 2:**
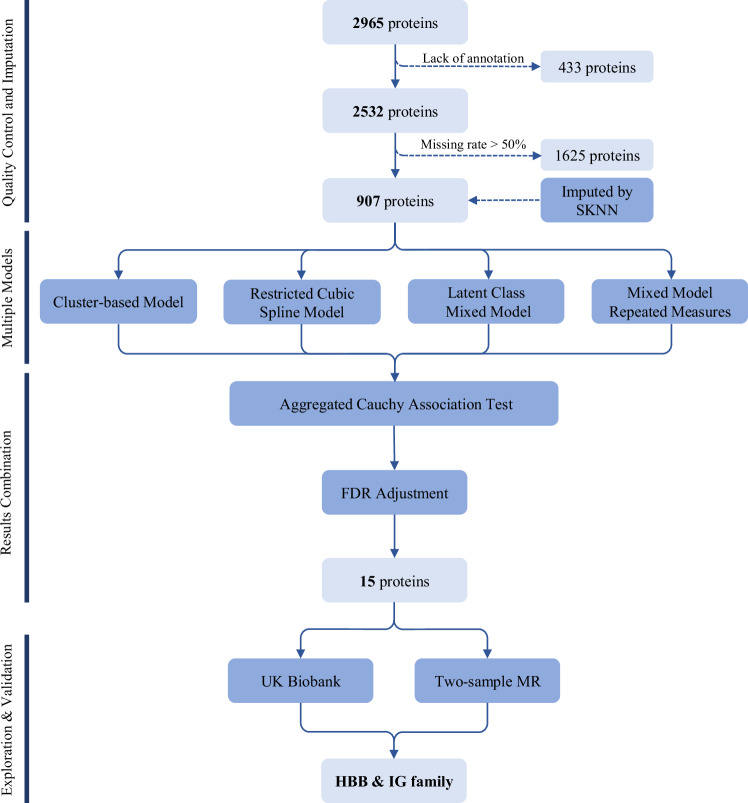
Flowchart of this study. Four steps were designed in this study: (1) Quality control and imputation for the protein levels; (2) Exploring the associations between proteins and FEV_1_ based on four distinct models; (3) Combining the results of each model using ACAT approach; (4) External exploration and validation using proteomic data from UK Biobank and two-sample Mendelian Randomization. Notes: FEV_1_ forced expiratory volume in one second, ACAT aggregated Cauchy association test, FDR false discovery rate, MR Mendelian randomization, HBB Hemoglobin subunit beta, IG Immunoglobulin.

Owing to the limited sample size and the extensive number of proteins under investigation, conventional statistical analysis methods, a linear regression analysis between the decline rate of FEV_1_ and proteins, proved inadequate, i.e., no significant associations were discovered between the decline rate of FEV_1_ and any protein after multiple correction (Fig. S1). Consequently, a multi-steps strategy was employed to explore the relationship between proteins and lung function (see workflow in Fig. 2).

In the first step, recognizing that the associations between long-term lung function trends and protein levels is not merely linear, we utilized four distinct models to assess the relationship between proteins and FEV_1_, including cluster-based model, restricted cubic spline (RCS) model, latent class mixed model (LCMM), and mixed model for repeated measurements (MMRM). A comprehensive description of all these models can be found in the supplementary methods. In all of the models, we incorporated adjustments for the same covariates, including age, height, gender, cumulative pack-years of smoking, log-transformed cumulative endotoxin exposure with silk workers set at zero, and years since retirement.

In the second step, we combined the *P*-values obtained from four models for each protein, using aggregated Cauchy association test (ACAT) [26], which is a robust method for combining *P*-values, accommodating arbitrary dependency structures. The combined *P*-values were adjusted for false discovery rate (FDR) using the Benjamini–Hochberg method [27], with statistical significance threshold defined as FDR-*q* < 0.05. The ACAT method was generated in R package *ACAT*.

In the validation study, we investigated the association between the rate of decline in FEV_1_ and protein using linear regression analysis. The rate of decline in FEV_1_ was determined as the slope of a linear regression model fitted to all reproducible FEV_1_ measurements plotted against age for each sample. The model was adjusted for baseline age, sex, height, pack-years of smoking, and baseline FEV_1_. The results were adjusted for FDR, and FDR-*q* values < 0.05 were considered significant.

### Protein-protein Interaction analysis

Due to the distinct proteomics assays in the Shanghai Textile Worker Study and UKB, resulting in a limited overlap of all proteins, protein-protein interaction (PPI) network analyses were conducted for proteins potentially associated with lung function from each cohort. These analyses were performed using the STRING database (version 12.0) [28].

### Mendelian randomization analysis

Finally, to validate further our findings and explore the causal relationship between proteins and lung function, we conducted two-sample MR analysis between significant proteins and FEV_1_. The genome-wide association study (GWAS) summary data of FEV_1_ was used from UK Biobank Neale Lab (http://www.nealelab.is/uk-biobank), comprising 361,194 samples from the United Kingdom. The GWAS summary data was adjusted for age, age^2^, sex, age × sex, age^2 ^× sex, and 20 genetic principal components. The protein quantitative trait loci (pQTL) database utilized in our study was derived from the AGES cohort of 5368 elderly Icelanders [29], which performed a GWAS involving 4782 serum proteins. For our main analysis, we used inverse variance weighted method (IVW). Additionally, several alternative MR methods under different assumptions were also performed as sensitivity analysis to further validate our results: (i) MR-Egger [30]: this method relies on Instrument Strength Independent of Direct Effect assumption and can provide the causal effect estimate as well as test for pleiotropy; (ii) MR-Pleiotropy Residual Sum and Outlier (MR-PRESSO) [31]: it is capable of identifying and removing outliers with horizontal pleiotropic effects; (iii) Generalized Summary-data-based Mendelian Randomization (GSMR) [32]: this method can account for linkage disequilibrium (LD) between instrumental variables, as well as detect and eliminate genetic instruments that have apparent pleiotropic effects on both exposure and outcome. The MR analysis conducted from FEV_1_ to proteins using standard methods followed a specific procedure to select independent instrumental variants. Initially, we identified the genome-wide significant single nucleotide polymorphisms (SNPs) with *P*-value < 5 × 10^–8^, then 1000 Genomes Project phase 3 of European population served as the LD reference panel to obtain independent instrumental variables with *r*^2^ < 0.001 or physical distance >10,000 kb. In the inverse analysis from proteins to FEV_1_, the significance threshold was relaxed to *P*-value < 5 × 10^–6^, due to independent instrumental variables. We employed *F* statistics to assess the strength of genetic associations of instrumental variables and the issue of weak instrument bias. MR analysis was performed using R packages *TwoSampleMR* and *GSMR*.

All statistical analyses were performed using R software (version 4.2.0).

## Results

### Population characteristics

A total of 453 subjects participated in the 2016 survey, with 40 excluded due to invalid data or low quality of serum samples. Characteristics of the 413 subjects are presented in Table 1. All subjects had retired from active textile work, with an average retirement year of 24.5 ± 4.7 years, and the average age was 70.8 ± 8.7 years old. Apart from the years since retirement, the characteristics of cotton and silk workers were generally comparable. Regarding lung function, there were no significant differences in mean FEV_1_ and FVC between cotton and silk workers in the last survey. However, the annual decline in FEV_1_ was greater among cotton workers compared to silk workers (25.2 vs. 22.5 ml/year, *P* = 0.012).

**Table 1:** Characteristics of study population in the last survey (2016).

	Cotton workers (*n* = 221)	Silk workers (*n* = 192)	*P*
Sex, *n*(%)			0.465
Male	73 (33.0%)	57 (29.7%)	
Female	148 (67.0%)	135 (70.3%)	
Age (years)	69.8 ± 8.7	70.3 ± 8.6	0.567
Height (cm)	162 ± 7.7	161 ± 7.4	0.100
Smoking status, *n*(%)			0.456
Non-smoker	155 (70.1%)	145 (75.5%)	
Current	34 (15.4%)	23 (12.0%)	
Former	32 (14.5%)	24 (12.5%)	
Pack-years^a^	36.0 ± 29.0	30.0 ± 19.6	0.213
Years since retirement (years)	24.0 ± 5.1	25.0 ± 4.1	0.018
Cumulative endotoxin exposure (EU/m^3^-years)	45,930.0 ± 39,954.9	–	–
Baseline FEV_1_ (ml)^b^	2980.4 ± 718.1	2853.6 ± 672.1	0.065
FEV_1_ (ml)	2089.7 ± 594.5	2088.3 ± 546.9	0.981
FVC (ml)	2756.8 ± 738.3	2771.8 ± 722.0	0.841
FEV_1_/FVC (%)	75.7 ± 6.5	75.6 ± 7.3	0.852
% predicted FEV_1_ (%)^c^	101.3 ± 18.1	104.3 ± 18.5	0.117
Annual FEV_1_ decline (ml/year)^d^	25.2 ± 11.0	22.5 ± 9.6	0.012
Any respiratory symptoms	75 (33.9%)	77 (40.1%)	0.195
Chronic bronchitis	15 (6.8%)	14 (7.3%)	0.841
Dyspnea	67 (30.3%)	69 (35.9%)	0.225

The chi-square test was used for categorical variables and *t*-test for continuous variables.

*FEV*_*1*_ forced expiratory volume in one second, *FVC* forced vital capacity.

^a^Calculated among ever-smokers only.

^b^Obtained from baseline survey at 1981.

^c^Calculated using updated Global Lung Function Initiative (GLI) equations.

^d^Calculated by taking the difference in FEV_1_ between 2016 and 1981 divided by elapsed time.

### Protein associations with lung function in separate models

In each of the four distinct models applied to 907 proteins following quality control, we tested potential associations between different proteins and FEV_1_. However, for the majority of these associations, statistical significance remained unattained after adjusting for multiple testing. In the clustering model, we identified that four clusters exhibited the optimal performance, as determined by within cluster sum of squares less than 20% (Figs. S2 and 3 and Table S1). Besides, there were significant differences in gender and smoking status across clusters (Table S2). Subsequently, upon incorporating these clusters into the outcome model, we identified 29 proteins exhibiting potential significant associations (*P* < 0.05) (Fig. S4). In the case of the RCS model, we identified 39 proteins with a potential linear or non-linear association with FEV1 (*P* < 0.05) (Fig. S5). Within the framework of LCMM for trajectory analysis, we categorized the FEV_1_ trajectories into four distinct subgroups based on criteria such as the Akaike Information Criterion, the Bayesian Information Criterion, and entropy, while ensuring that none of these subgroups had an insufficient sample size (less than 5%) (Table S3 and Figs. S6 and 7). By integrating the FEV_1_ trajectory subgroups into the LCMM model, we identified 86 proteins that exhibited potential associations with lung function trajectories (*P* < 0.05) (Fig. S8). However, none of these associations from the three models above survived FDR adjustment. Last, using MMRM, we identified 182 proteins which showed a potential association with FEV_1_ (*P* < 0.05), while 39 proteins passed FDR adjustment (Figs. S9 and 10).

### Combined protein associations with lung function

The unadjusted *P*-values obtained from the four models above were combined using the ACAT approach and subsequently adjusted for FDR. This comprehensive analysis revealed associations between 15 proteins and FEV_1_ (see Table 2 for details). Among these proteins, two were subunits of hemoglobin, specifically hemoglobin subunit beta (HBB, FDR-*q*_ACAT_ = 0.040), alpha globin chain (HBA2, FDR-*q*_ACAT_ = 0.045). In addition, four proteins were identified as fragments of immunoglobulins, including four immunoglobulin subunits: immunoglobulin kappa variable 3–7 (IGKV3-7, FDR-*q*_ACAT_ = 0.003), and immunoglobulin heavy chain variable regions (IgH, FDR-*q*_ACAT_ = 0.011). The results of proteomic data imputed by minimum values were generally consistent with the main results and were presented in Table S4 for sensitivity analysis.

**Table 2:** Proteins associated with long-term decline of FEV_1_ in Shanghai Textile Worker Study.

Protein	Gene	*P*_ACAT_	FDR-*q*_ACAT_	Function^a^	Publication evidence of association with lung function^b^
Epididymis luminal protein 180	HEL180	1.05 × 10^–6^	7.40 × 10^–4^	Adaptive immunity	
Coiled-coil domain-containing protein 80	CCDC80	1.63 × 10^–6^	7.40 × 10^–4^	Fibronectin binding	[43]
immunoglobulin kappa variable 3–7	IGKV3-7	8.81 × 10^–6^	2.66 × 10^–3^	Adaptive immunity	[40]
Solute carrier family 25 member 3	SLC25A3	4.77 × 10^–5^	1.08 × 10^–2^	Phosphate ion transport	
Ig heavy chain variable region	IgH	6.44 × 10^–5^	1.11 × 10^–2^	Adaptive immunity	
Bone morphogenetic protein 1	BMP1	7.33 × 10^–5^	1.11 × 10^–2^	Growth factor	[44]
Protein-lysine 6-oxidase	LOX	1.60 × 10^–4^	2.08 × 10^–2^	Metal binding	[45]
Ig heavy chain variable region	IgH	3.22 × 10^–4^	3.64 × 10^–2^	Adaptive immunity	
Putative uncharacterized protein CLLU1-AS1	CLLU1-AS1	3.83 × 10^–4^	3.86 × 10^–2^	Chronic lymphocytic leukemia	
Anti-folate binding protein	HuVH8B VH	4.78 × 10^–4^	4.04 × 10^–2^	Adaptive immunity	
Hemoglobin subunit beta	HBB	4.91 × 10^–4^	4.04 × 10^–2^	Oxygen transport	
Ig heavy chain variable region	IgH	6.47 × 10^–4^	4.54 × 10^–2^	Adaptive immunity	
Alpha globin chain	HBA2	6.73 × 10^–4^	4.54 × 10^–2^	Oxygen transport	
Insulin-like growth factor-binding protein 4	IGFBP4	7.01 × 10^–4^	4.54 × 10^–2^	Growth factor binding	[46]
Alpha globin chain	HBA2	7.63 × 10^–4^	4.54 × 10^–2^	Oxygen transport	

Protein and gene names are retrieved from UniProt database.

*ACAT* aggregated Cauchy association test, *FDR* false discovery rate.

^a^Protein functions were retrived from Uniprot database.

^b^Potential association discovered from published studies.

### Protein associations in the exploratory study

**Fig. 3:**
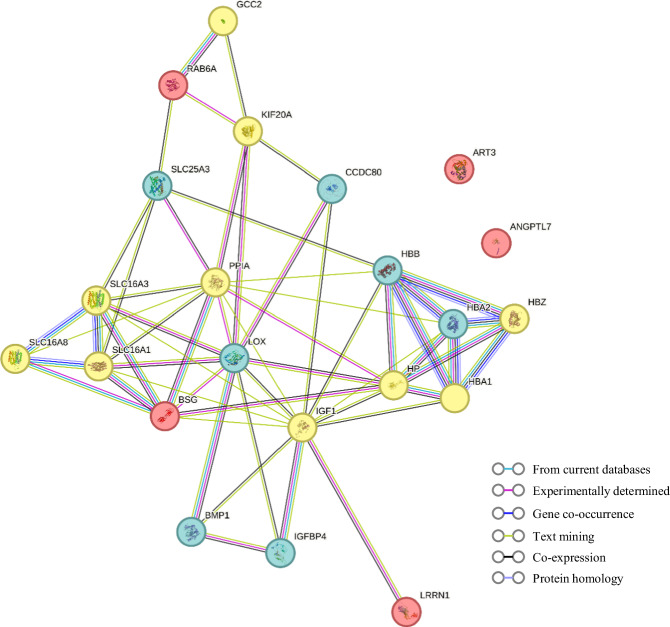
Protein-protein interaction network of potential protein markers in Shanghai Textile Worker Study and UK Biobank. Blue circles represent potential protein markers identified through the Shanghai Textile Workers Study, red circles represent potential protein markers identified through UKB, and yellow circles are transition proteins associated with potential biomarkers identified in both cohorts.

Among the 2923 independent proteins in UKB, five exhibited significant associations with the rate of FEV_1_ decline following FDR correction. These proteins include ART3, RAB6A, LRRN1, ANGPTL7, and BSG (Table 3 and Fig. S11). In the PPI network analysis, RAB6A, LRRN1, and BSG were found to be associated with potential protein markers identified in the Shanghai Textile Workers Study to varying degrees (Fig. 3). Especially, associations can be observed among HBB proteins and BSG, RAB6A, and LRRN1, facilitated by relationships such as co-expression with proteins like HP, SLC25A3, and IGF1, respectively.

**Table 3:** Proteins associated with the rate of decline of FEV_1_ in UK Biobank.

Protein	Gene	*β*	*se*	*P*	FDR-*q*
Ecto-ADP-ribosyltransferase 3	ART3	2.64 × 10^–5^	5.56 × 10^–6^	2.11 × 10^–6^	6.16 × 10^–3^
Ras-related protein Rab-6A	RAB6A	1.69 × 10^–5^	4.09 × 10^–6^	3.66 × 10^–5^	3.14 × 10^–2^
Leucine-rich repeat neuronal protein 1	LRRN1	1.95 × 10^–5^	4.75 × 10^–6^	4.23 × 10^–5^	3.14 × 10^–2^
Angiopoietin-related protein 7	ANGPTL7	2.05 × 10^–5^	5.05 × 10^–6^	5.28 × 10^–5^	3.14 × 10^–2^
Basigin	BSG	3.72 × 10^–5^	9.21 × 10^–6^	5.38 × 10^–5^	3.14 × 10^–2^

### Protein associations using Mendelian randomization

To validate further our findings, we conducted a two-sample MR analysis on significant associations identified previously. Among the 15 proteins significantly associated with FEV_1_, a total of three of them were successfully matched from the pQTL database, including CCDC80, HBB, and IGFBP4. The results of bi-directional MR analysis, employing the inverse-variance weighted method, revealed a bi-directional correlation between HBB and FEV_1_. Specifically, a positive correlation was observed from FEV_1_ to HBB, while a negative correlation existed from HBB to FEV_1_ (Table 4 and Figs. S12 and 13). Various alternative MR methods produced largely consistent results. For the other proteins, the MR analysis did not yield significant results (Table S5 and 6). Additionally, as four of the 15 significant proteins belonged to the immunoglobulin family, we conducted a bidirectional MR analysis encompassing all immunoglobulin family proteins from the pQTL database. The results, based on the inverse-variance weighted method, indicated that among the nine proteins in the immunoglobulin family from the pQTL database, four showed significant associations with FEV_1_. These associations were largely characterized by negative relationships from FEV_1_ to the proteins, with one protein exhibiting a significant negative association in both directions (Table S7 and 8).

**Table 4:** Two-sample Mendelian randomization of HBB.

Method	No. SNPs	beta	SE	*P*
FEV_1_ to HBB				
IVW	206	–0.213	0.092	0.018
MR-Egger	206	0.013	0.300	0.966
MR-PRESSO	206	–0.218	0.092	0.019
GSMR	206	–0.238	0.093	0.011
HBB to FEV_1_				
IVW	17	0.015	0.006	0.019
MR-Egger	17	0.040	0.014	0.014
MR-PRESSO	17	0.015	0.006	0.032
GSMR	17	0.014	0.005	0.008

*HBB* hemoglobin subunit beta, *FEV*_*1*_ forced expiratory volume in one second, *No. SNPs* number of single nucleotide polymorphisms, *SE* standard error, *IVW* inverse-variance weighted, *MR* Mendelian randomization, *MR-PRESSO* MR-pleiotropy residual sum and outlier, *GSMR* generalized summary-data-based Mendelian randomization.

## Discussion

The Shanghai Textile Worker Study cohort had a total of seven follow-ups on lung function over a 35-year period, which is one of the longest follow-ups and most frequent lung function measurements in an occupational population. Through this large-scale proteomics study of the longest-follow-up pulmonary function cohort of textile workers to date, we identified multiple novel proteins associated with long-term decreasing trends in FEV_1_ through the combined results of multiple models and validated them by two-sample Mendelian randomization. Our findings fully utilized the lung function trajectory information and provide new potential biomarkers associated with long-term decline in lung function for occupational populations in China.

This large-scale proteomics study faced limitations stemming from sample size, resulting in insufficient statistical power for conventional methods. Nevertheless, leveraging the large number of repeated FEV_1_ measurements, we overcame this challenge by using a strategy of multiple modeling, targeting various facets of FEV_1_ trajectories, which is the major strength of this study. Specifically, the clustering model, centered on FEV_1_ baselines and slopes, primarily emphasized linear features, allowing for the identification of less significant associations. In the meantime, the RCS model considered nonlinear effects in the relationships between FEV_1_ and proteins. The results of RCS models also suggested the presence of potential nonlinear associations for selected proteins. Employing the LCMM, a classical model for trajectory analysis, we effectively discerned long-term FEV_1_ trajectories into several subgroups, and obtained superior association assessment performance than the previous two models. Finally, we reversed the causal relationship between FEV_1_ and proteins using reverse regression and applied a MMRM, which utilized fully the multiple repeated measurements of FEV_1_ and achieved optimal test. Using the ACAT method, we combined outcomes from four distinct models that captured various facets of the FEV_1_ trajectories in a robust manner. These results represented the collective correlation of proteins with diverse facets of FEV_1_ trajectories. It is important to note that these correlations do not imply a straightforward causal relationship. Nevertheless, our methodology is enlightening, particularly for studies characterized by low number of subjects and multiple comparisons.

One of the advantages of applying UKB data is the sufficient sample size. In that case, we used UKB as an independent exploratory study. However, due to numerous heterogeneities in the populations such as different ethnicities, occupations, protein detection platforms etc., the analysis of UKB still could not serve as an independent validation of the Shanghai Textile Worker Study. Nevertheless, potential associations between the proteins identified through UKB and the Shanghai Textile Worker Study can still provide supporting evidence for our findings. For example, RAB6A (Member RAS Oncogene Family) was found significantly associated with FEV_1_ in UKB, while it has been proved to be co-expressed with SLC25A3, which enhanced our results. Moreover, BSG, i.e., basigin, could be associated with hemoglobin like HBB and HBA2 through the connection of HP (haptoglobin).

Given that the serum samples were gathered subsequent to all repeated measurements of lung function in this study, it poses a challenge to investigate the causal relationship between FEV1 and proteins. Consequently, we implemented MR analysis to externally validate potential associations and causal evidence linking protein biomarkers to lung function. IVW method is generally used as the main outcome in MR analysis. A significant advantage of this method is its statistical efficiency and precision [33]. MR-Egger method has a better performance when there are uncorrelated pleiotropic effects of the genetic variants. However, the causal estimate using MR-Egger method may be biased and have inflated type-1 error under other circumstances [34]. Our findings suggest a significant association between several hemoglobin subunits and long-term declines in FEV_1_. Additionally, MR analysis revealed a bidirectional causal relationship between HBB and FEV_1_. Specifically, FEV_1_ exhibits a negative correlation with HBB proteins, while HBB shows a positive correlation with FEV_1_.

As a subunit of hemoglobin, HBB is expected to be predominantly present in erythrocytes. However, its presence in serum is not uncommon, attributable to the erythrocyte cell cycle and potential hemolytic reactions. In a comparative analysis of the proteomes of erythrocytes, platelets, whole blood, and plasma, no correlation was observed between the erythrocyte proteome and the plasma proteome [35]. However, HBB was consistently detected in pure plasma across most individuals, indicating the presence of HBB in both plasma and serum, not solely attributable to erythrocyte hemolysis [36]. Hemoglobin has also previously been found to link to acute lung inflammation. For instance, a GWAS investigating pneumonia susceptibility and severity demonstrated a significant impact of the rs334 locus within the HBB gene [37]. Additionally, sickle cell anemia, caused by the substitution of valine for glutamate in the sixth amino acid of the HBB protein, has been identified through epidemiological studies as an independent risk factor for pneumonia [38]. Furthermore, proteomic analysis of airway mucus from severe COVID-19 patients revealed variations in the expression of HBB and HBA1 proteins [39]. Collectively, these studies suggest a complex regulatory relationship between lung inflammation and hemoglobin. This intricate interplay may further impact the association between HBB and the long-term decline in lung function. Nevertheless, our study is the first to establish a connection between hemoglobin and the prolonged decline in lung function.

The humoral immune response mediated by B cells plays a crucial role in regulating acute infections. Numerous studies have investigated the immune response to acute infections of the lung. In particular, two independent studies revealed significant upregulation of IGKV3-7 in asymptomatic COVID-19 individuals [40] and IGLV9-49 in COVID-19 patients [41] respectively, which implies that these two proteins may be involved in acute lung inflammation. In textile workers, this protein may also be associated with exposure to exogenous dust and bacteria endotoxin. Our study is the first to associate immunoglobulins with long-term decline of lung function, potentially mediated by acute lung inflammation. This contributes to our comprehension of the biological mechanisms responsible for the long-term decline of lung function.

We acknowledge several limitations of this study. First, since protein levels were assessed during the final follow-up in 2016, subsequent to all repeated lung function measurements, we are unable to establish a definitive causal relationship between lung function and protein levels. Earlier research of this cohort has indicated that lung inflammation, caused by cotton dust and bacteria endotoxin, may contribute to sustained lung function deterioration, but it remains unclear whether the specific protein under investigation functions as a mediator in this process. Employing MR analyses could offer preliminary insights into the directional causal relationships between proteins and lung function. For example, in our study, the decline of lung function may positively regulate hemoglobin levels, which in turn may exerting a positive effect on lung function. Our research aims to identify potential protein biomarkers linked to long-term decline in lung function. To validate significant associations, further studies are necessary to corroborate the underlying biological mechanisms, such as the causal relationship and biological mechanisms between hemoglobin, including HBB and HBA2, and FEV_1_.

Second, given that our entire study population survived until the last follow-up in 2016, the presence of a healthy worker survivor effect is inevitable. Approximately 10% of our study cohort met the spirometry criteria for COPD, and the longitudinal change in FEV_1_ was comparatively smaller within our study group in contrast to the general population. These factors may influence the extrapolation of our findings. Nonetheless, our study’s objective was to investigate protein biomarkers linked to long-term decline in lung function in occupational populations, a context where the influence of healthy worker survivor effect may not be substantial. Our findings were validated through a two-sample MR analysis conducted within the general population, which would also mitigate the impact of the healthy worker survivor effect. Moreover, the presence of a healthy worker confounding would bias our results towards to null hypothesis. In the meantime, almost every worker has been retired for more than twenty years at the time of serum sampling. Although the genuine effect of retirement remains unknown, we considered it as a covariate for the association of proteins and FEV_1_, which may not lead to biased results of this study.

Third, the Shanghai Textile Workers Cohort consists exclusively of Han Chinese participants. However, our results lack validation within a Chinese population, which may affect the generalizability of our findings. A recent large-scale serum proteomic investigation in a Chinese cohort analyzed a total of 304 proteins [42], yet failed to match the proteins we found to be potentially associated with the decline of lung function. MR analyses conducted with European populations could only confirm more conservative associations spanning different ethnicities. While this enhances the robustness of our findings, it may also constrain the generalizability of our results. Moreover, comparing with serum proteomics, proteomic studies from respiratory tract, such as bronchoalveolar lavage proteomics, could be more useful in assessing lung function decline, which relies on further research. Nonetheless, this study sets a precedent for large-scale proteomic studies of lung function decline in textile workers.

## Conclusion

In summary, through a large-scale proteomics study of the longest-follow-up pulmonary function cohort of textile workers to date, we identified multiple novel proteins, including hemoglobin beta subunit and subunits of immunoglobulins, associated with long-term decreasing trends in FEV_1_. These associations were confirmed through the aggregation of results from multiple models and further validated using a two-sample MR approach. Our findings provide new potential biomarkers associated with long-term decline in lung function for occupational populations in both China and Europe. The causal direction of the relationship between proteins and lung function, along with the underlying biological mechanisms, such as the pathway between hemoglobin and FEV_1_, remain uncertain, necessitating further research.

## Supplementary information

Supplemental material

## Data Availability

Data are accessible upon reasonable request made to the corresponding authors.

## Code availability

Codes generated and used in this study have been uploaded into GitHub: https://github.com/WeiLab4Research/Cotton_Proteomics.
