# Supplementary material for: Proteomic biomarkers of long-term lung function decline in textile workers: a 35-year longitudinal study
Source: J Expo Sci Environ Epidemiol. 2024 Oct 2;35(4):602–10. doi: 10.1038/s41370-024-00721-7 (PMC12234346; doi:10.1038/s41370-024-00721-7)
Supplement: Supplementary file 1 — Supplemental material [file 41370_2024_721_MOESM1_ESM.docx]

**Supplemental Material**

**Title: Proteomic Biomarkers of Long-term Lung Function Decline in Textile Workers: A 35-Year Cohort Study**

Mengsheng Zhao et., al.

**Table of Contents**

**Supplemental Methods**

**Supplemental Tables and Figures**

- **Table S1.** Baseline and slope of FEV_1_ trajectories in each cluster (Mean ± SD).
- **Table S2.** Basic characteristics in each cluster (%).
- **Table S3.** Model fit parameters for latent class mixed models with 1-6 classes.
- **Table S4.** Proteins associated with long-term decline of FEV1 in Shanghai Textile Worker Study imputed by minimum values for sensitivity analysis.
- **Table S5.** Two-sample Mendelian randomization analysis for proteins showing significant associations identified in our study, excluding HBB (FEV_1_ to proteins).
- **Table S6.** Two-sample Mendelian randomization analysis for proteins showing significant associations identified in our study, excluding HBB (proteins to FEV_1_).
- **Table S7.** Two-sample Mendelian randomization for all immunoglobulin family proteins (FEV_1_ to proteins).
- **Table S8.** Two-sample Mendelian randomization for all immunoglobulin family proteins (proteins to FEV_1_).
- **Figure S1.** Volcano plot for associations between decline rate of FEV_1_ and proteins.
- **Figure S2.** Elbow plot for K-means clustering.
- **Figure S3.** Average linear trajectories of FEV_1_ in each cluster. Note: FEV_1_: forced expiratory volume in one second.
- **Figure S4.** Volcano plots of cluster-based model (cluster2 as reference cluster), showing the gene name of top 5 significant proteins, where dashed line represents the nominal significant level (*P* < 0.05).
- **Figure S5.** Volcano plots of restricted cubic spline model, showing the gene name of top 5 significant proteins, where dashed line represents the nominal significant level (*P* < 0.05).
- **Figure S6.** Average and individual (gray) trajectories of FEV_1_ in each latent class using latent class mixed model.
- **Figure S7.** Summary of average trajectories of FEV_1­_ in each latent class using latent class mixed model.
- **Figure S8.** Volcano plots of latent class mixed model (class1 as reference cluster), showing the gene name of top 5 significant proteins, where dashed line represents the nominal significant level (*P* < 0.05).
- **Figure S9.** Volcano plots of mixed model for repeated measurements, with protein effects at specific ages, showing the name of top 5 significant proteins, where two dashed lines represent the nominal significant level (*P* < 0.05) and the FDR-corrected significant level (FDR-*q* < 0.05), respectively.
- **Figure S10.** Volcano plot of mixed model for repeated measurements, with the effects of protein*age interactions, showing the name of top 5 significant proteins, where two dashed lines represent the nominal significant level (*P* < 0.05) and the FDR-corrected significant level (FDR-*q* < 0.05), respectively.
- **Figure S11.** Volcano plot of proteomic from UK Biobank in exploratory study, showing the name of top 5 significant proteins, where two dashed lines represent the nominal significant level (P < 0.05) and the FDR-corrected significant level (FDR-q < 0.05), respectively.
- **Figure S12.** Scatter plot of two-sample Mendelian randomization (HBB to FEV_1_).
- **Figure S13.** Scatter plot of two-sample Mendelian randomization (FEV_1_ to HBB).

## **Supplemental Methods**

***Proteomics Profiling***

**1 Protein extraction**

SDS free lysate was added to 100μL serum sample, and finally to make up a total volume of 1mL; then the proteins were reduced and alkylated to disrupt the disulfide bonds as following:

1) DTT was added to sample with a final concentration of 10 mM, and incubate at 37°C for 30 minutes;

2) Iodoacetamide was added to sample with a final concentration of 55mM, and incubated in the dark at room temperature for 30 minutes.

3) The mixture of proteins would be passed through a solid phase extraction (SPE) C18 column for protein enrichment.

**2 Protein enrichment**

1) Activation: The SPE C18 column was activated by methanol with a volume of 1mL at a rate of 3 drops per second.

2) Condition: The solution of 0.1% FA in 1mL was used to condition the SPE C18 column.

3) Sample loading: The diluted serum protein mixtures were loaded onto and passed through the SPE C18 column at a rate of 1 drop per second. If the volume of the mixture was more than 1mL, please repeat the operation for 2-3 times.

4) Washing: The solution of 0.1% FA in 3mL was used to washing those non-specific binding proteins with the SPE C18 column;

5) Elution: The enriched proteins were eluted with 800L 75% ACN at a rate of 0.5 drops per second.

6) Drying: The eluate was frozen and dried for further treatment.

**3 Protein enrichment quality control**

1) Protein quantification: The dried proteins were redissolved in a solution of 20μL 50mM ammonium bicarbonate. The protein solution was quantified according to the *Pierce Quantitative Fluorometric Peptide Assay* instructions.

2) SDS-PAGE: Each sample was taken with appropriate amount of protein solution and mixed with appropriate amount of sample buffer, heated at 95°C for 5 minutes, centrifuged at 25,000g for 5 minutes, and the supernatant was loaded into a well of a 12% SDS polyacrylamide gel, 80V constant pressure electrophoresis for 30 minutes followed by 120V constant pressure electrophoresis for 120 minutes. After electrophoresis, the gel was stained and de-stained by a protein staining instrument for 10 minutes and the images were scanned.

**4 Protein digestion**

Trypsin was added to the protein solution with a ratio of 1:20 for enzyme and proteins (w/w). The mixture was incubated for 14-16h at 37°C.

**5 High pH RP separation**

An equal number of peptides from each sample was taken to pool into a mixture, and 20μg of mixture was diluted with 2mL of mobile phase A (5% ACN pH 9.8) for injection. The Shimadzu LC20AB HPLC system coupled with a Gemini high pH C18 column (5m, 4.6 x 250mm) was used. The sample was subjected to the column and then eluted at a flow rate of 1 mL/min by gradient: 5% mobile phase B (95% ACN, pH 9.8) for 10 minutes, 5% to 35% mobile phase B for 40 minutes, 35% to 95% mobile phase B for 1 minute, flow Phase B lasted 3 minutes and 5% mobile phase B equilibrated for 10 minutes. The elution was collected every 1 minute. Finally, all the fractions were combined into a total of 10 fractions, which were then frozen and dried.

**6 DIA analysis by nano-LC-MS/MS**

The dried peptide samples were reconstituted with mobile phase A (2% ACN, 0.1% FA), centrifuge at 20,000g for 10 minutes, and the supernatant was taken for injection. Separation was carried out by a Thermo UltiMate 3000 UHPLC liquid chromatograph. The sample was first enriched in the trap column and desalted, and then entered a tandem self-packed C18 column (150μm internal diameter, 1.8μm column size, 35cm column length), and separated at a flow rate of 500nL/min by the following effective gradient: 0~5 minutes, 5% mobile phase B (98% ACN, 0.1% FA); 5~90 minutes, mobile phase B linearly increased from 5% to 25%; 90~105 minutes, mobile phase B rose from 25% to 35%; 105~110 minutes, mobile phase B rose from 35% to 80%; 110~115 minutes, 80% mobile phase B; 115~120 minutes, 5% mobile phase B. The nanoliter liquid phase separation end was directly connected to the mass spectrometer as the following settings.

For DIA analysis, LC separated peptides were ionized by nanoESI and injected to tandem mass spectrometer Q-Exactive HF X (Thermo Fisher Scientific, San Jose, CA) with DIA (data-independent acquisition) detection mode. The main settings were: ion source voltage 1.9~2kV; MS scan range 400~1,250m/z; MS resolution 120,000, MIT 50ms; 400~1,250m/z was equally divided to 45 continuous windows MS/MS scan. MS/MS collision type HCD, MIT was auto mode. Fragment ions were scanned in Orbitrap, MS/MS resolution 30,000, collision energy was distributed mode: 22.5, 25, 27.5, AGC was 1E6.

***Statistical Analysis***

Owing to the limited sample size and the extensive number of proteins under investigation, conventional statistical analysis methods proved inadequate. Consequently, this study employed a three-step strategy to explore the relationship between proteins and lung function. In the first step, we utilized four distinct models to assess the relationship between proteins and FEV_1_, including cluster-based model, restricted cubic spline (RCS) model, latent class mixed model (LCMM), and mixed model for repeated measurements (MMRM).

**Cluster-based model**

Considering the predominantly linear feature of individual FEV_1_ trajectories, we performed a decomposition of individual FEV_1_ into two distinct attributes: baseline and slope. Subsequently, we applied the K-means clustering technique to these quantitative metrics. We determined the optimal number of clusters by assessing the within-cluster sum of squares (less than 20%) and examining an elbow plot. Following the clustering of individual FEV_1_ baseline and slope attributes, we incorporated the clusters as dummy variables into **Model 1**, which is presented below. Once a reference group had been chosen, we utilized the outcomes from the group exhibiting the more pronounced effect as an indicator of the protein's significance.

**Model 1:**

$$Protein=\alpha+\sum_{k=1}^{K-1} \beta_{k} Cluster_{k}+\boldsymbol{X\beta}+\varepsilon[1]$$

where $\alpha$ is a constant, $K$ represents the number of clusters, $\beta_{k}$ is the coefficient of $Cluster_{k}$ relative to the reference cluster, $\boldsymbol{X}$ represents a vector of all covariates, including sex, height, age, cumulative pack-year of smoking, log-transformed cumulative endotoxin exposure, and years since cessation, while $\boldsymbol{\beta}$ **­**is the vector of coefficients corresponding to these covariates, and $\varepsilon$ stands for a random error.

**Restricted cubic spline model**

Recognizing the potential nonlinearity in the influence of long-term trends in lung function on proteins, we employed restricted cubic splines (RCS) to model the slope of lung function decline. RCS is a popular way to explore the nonlinear relationship in regression models flexibly and is widely used in epidemiology and clinical trials. (Harrell et al., 1988) This allowed us to assess the distinct linear and nonlinear effects of the long-term rate of lung function decline on proteins. Subsequently, we constructed **Model 2**, which is presented in the following section. We used three knots in the RCS model. The stronger of the linear and nonlinear effects was employed as an indicator of protein significance.

**Model 2:**

$$Protein=\alpha+\beta_{1}*rcs\left( slope,3 \right)+\beta_{2}\boldsymbol{*}baseline+\boldsymbol{X\beta}+\varepsilon[2]$$

where $slope$ represents the slope of individual FEV_1_ trajectories, $baseline$ represents the baseline of individual FEV_1_ trajectories, while $\beta_{1}$ and $\beta_{2}$ represent their coefficients, with the remaining symbols as previously mentioned.

**Latent class mixed model**

To analyze long-term trajectories of individual FEV1, we employed the latent class mixed model (LCMM), a commonly used approach in trajectory analysis. This method allowed us to categorize study participants into distinct latent trajectory classes. We fitted models with varying numbers of latent classes, ranging from one (representing no latent class) to six. The membership probabilities for each participant within these trajectory classes were estimated using the maximum probability assignment rule. (Strauss et al., 2014) The optimal number of latent variables was determined by assessing the model fit metrics, such as the Akaike Information Criterion (AIC), the Bayesian Information Criterion (BIC), and entropy, while ensuring that the sample size in each latent class was not less 5456745than 5% of the total sample size. (Herle et al., 2020)

After determined the number of latent classes and assigned all participants into different classes, we incorporated the latent classes as dummy variables into **Model 3**, which is presented below. Once a reference class had been chosen, we used the outcomes from the class exhibiting the more pronounced effect as an indicator of the protein's significance.

**Model 3:**

$$Protein=\alpha+\sum_{l=1}^{L-1} \beta_{l} Class_{l}+\boldsymbol{X\beta}+\varepsilon[3]$$

where $L$ represents the number of latent classes, $\beta_{l}$ is the coefficient of $Class_{l}$ relative to the reference class, with the remaining symbols as previously mentioned.

**Mixed model for repeated measurements**

To harness the complete potential of repeated FEV_1_ measurements, we applied a reverse regression concept. In this approach, we reversed the causality in the regression equation, with 'proteins' as the dependent variable and 'repeated measurements of FEV_1_' as the independent variable. We employed this methodology to build a mixed-effects model for repeated measurements (MMRM). The model encompassed fixed protein levels, baseline FEV_1_, all relevant covariates, and interactions of protein-age and protein-endotoxin. Furthermore, a random intercept term was included, as specified in **Model 4**.

**Model 4:**

$$FEV1_{ij}=\alpha+\beta_{1}*protein_{i}+\beta_{2}*protein_{i}*age_{ij}+\beta_{3}*protein_{i}*endotoxin_{ij}+\beta4*baseline_{i}+\boldsymbol{X\beta}+\mu_{i}+\varepsilon_{ij} [4]$$

where $i$ represents distinct individuals, $j$ represents distinct repeated measurements, $endotoxin$ represents log-transformed cumulative endotoxin exposure, $\mu_{i}$ denotes a random intercept, $\varepsilon_{ij}$ denotes a random error. Covariates in the model include time-varying variables, including age, endotoxin, pack-years, and cessation years, along with time-fixed variables including height and sex.

Following the application of MMRM and considering the age distribution within the study population, we computed the total effect of each protein on FEV_1_ while maintaining a fixed age at 60, 70, 80, and 90 years, respectively. We determined the indicator of the protein's significance based on the more significant influence between the age-specific protein effect on FEV_1_ and the protein-age interaction effect on FEV_1_.

## References

Harrell, F. E., Jr., Lee, K. L., & Pollock, B. G. (1988). Regression models in clinical studies: determining relationships between predictors and response. *J Natl Cancer Inst*, *80*(15), 1198-1202. <https://doi.org/10.1093/jnci/80.15.1198>

Herle, M., Micali, N., Abdulkadir, M., Loos, R., Bryant-Waugh, R., Hübel, C., Bulik, C. M., & De Stavola, B. L. (2020). Identifying typical trajectories in longitudinal data: modelling strategies and interpretations. *Eur J Epidemiol*, *35*(3), 205-222. <https://doi.org/10.1007/s10654-020-00615-6>

Strauss, V. Y., Jones, P. W., Kadam, U. T., & Jordan, K. P. (2014). Distinct trajectories of multimorbidity in primary care were identified using latent class growth analysis. *J Clin Epidemiol*, *67*(10), 1163-1171. <https://doi.org/10.1016/j.jclinepi.2014.06.003>

## **Supplemental Tables**

**Table S1.** Baseline and slope of FEV_1_ trajectories in each cluster (Mean ± SD).

| Cluster | Samples | Baseline (ml) | *P* | Slope (ml/year) | *P* |
| --- | --- | --- | --- | --- | --- |
| 1 | 129 | 2237 ± 230 | <0.001 | -17 ± 7 | <0.001 |
| 2 | 47 | 4335 ± 428 |  | -33 ± 11 |  |
| 3 | 73 | 3463 ± 217 |  | -29 ± 11 |  |
| 4 | 164 | 2801 ± 166 |  | -21 ± 9 |  |

Note: FEV_1_: forced expiratory volume in one second.

**Table S2.** Basic characteristics in each cluster (%).

| Cluster | Samples | Worker | | *P* | Gender | | *P* | Smoking Status | | | *P* |
| --- | --- | --- | --- | --- | --- | --- | --- | --- | --- | --- | --- |
|  |  | Cotton | Silk |  | Male | Female |  | Non-smoker | Former | Current |  |
| 1 | 129 | 64 (49.6%) | 65 (50.4%) | 4.07×10^-2^ | 7 (5.4%) | 122 (94.6%) | <0.001 | 116 (90.0%) | 9 (7.0%) | 4 (3.0%) | <0.001 |
| 2 | 47 | 30 (63.8%) | 17 (36.2%) |  | 47 (100.0%) | 0 (0.0%) |  | 7 (14.9%) | 15 (31.9%) | 25 (53.2%) |  |
| 3 | 73 | 38 (52.1%) | 35 (47.9%) |  | 50 (68.5%) | 23 (31.5%) |  | 32 (43.8%) | 21 (28.8%) | 20 (27.4%) |  |
| 4 | 164 | 89 (54.3%) | 75 (45.7%) |  | 26 (15.9%) | 138 (84,1%) |  | 145 (88.4%) | 11 (6.7%) | 8 (4.9%) |  |

**Table S3.** Model fit parameters for latent class mixed models with 1-6 classes.

| Class | AIC | BIC | Entropy | C1% | C2% | C3% | C4% | C5% | C6% |
| --- | --- | --- | --- | --- | --- | --- | --- | --- | --- |
| 1 | 41865.5 | 41881.6 | 1.00 | 100.00 |  |  |  |  |  |
| 2 | 40947.3 | 40983.5 | 0.90 | 28.09 | 71.91 |  |  |  |  |
| 3 | 40731.4 | 40787.8 | 0.91 | 4.84 | 25.91 | 69.25 |  |  |  |
| 4 | 40648.1 | 40724.5 | 0.84 | 6.30 | 21.55 | 58.35 | 13.80 |  |  |
| 5 | 40586.6 | 40683.1 | 0.83 | 2.42 | 14.29 | 12.35 | 53.75 | 17.19 |  |
| 6 | 40565.1 | 40681.8 | 0.84 | 1.69 | 14.29 | 10.65 | 53.03 | 17.92 | 2.42 |

Note: AIC: Akaike information criterion; BIC: Bayesian information criterion; C*n*%: percentage of participants of class *n*.

**Table S4.** Proteins associated with long-term decline of FEV1 in Shanghai Textile Worker Study imputed by minimum values for sensitivity analysis.

| **Protein** | **Gene** | ***P*_ACAT_** | **FDR-*q*_ACAT_** |
| --- | --- | --- | --- |
| Coiled-coil domain-containing protein 80 | CCDC80 | 5.66×10-6 | 0.005 |
| immunoglobulin kappa variable 3-7 | IGKV3-7 | 2.05×10-5 | 0.007 |
| Epididymis luminal protein 180 | HEL180 | 2.16×10-5 | 0.007 |
| Hemoglobin subunit delta | HBD | 6.00×10-5 | 0.014 |
| Leucine-tRNA ligase | LARS1 | 1.30×10-4 | 0.023 |
| Ig heavy chain variable region | IgH | 1.85×10-4 | 0.023 |
| Protein-lysine 6-oxidase | LOX | 2.18×10-4 | 0.023 |
| Ig heavy chain variable region | IgH | 2.29×10-4 | 0.023 |
| Immunoglobulin lambda variable 9-49 | IGLV9-49 | 2.70×10-4 | 0.023 |
| Ceruloplasmin | CP | 2.80×10-4 | 0.023 |
| Ig heavy chain variable region | IgH | 2.85×10-4 | 0.023 |
| Guanylin | GUCA2A | 3.01×10-4 | 0.023 |
| Anti-folate binding protein | HuVH8B VH | 4.78×10-4 | 0.033 |
| Alpha globin chain | HBA2 | 6.66×10-4 | 0.042 |
| Insulin-like growth factor-binding protein 4 | IGFBP4 | 7.01×10-4 | 0.042 |
| Double-stranded RNA-specific adenosine deaminase | ADAR | 7.49×10-4 | 0.043 |
| Hemoglobin subunit beta | HBB | 8.85×10-4 | 0.047 |

Protein and gene names are retrieved from UniProt database. Note: ACAT, aggregated Cauchy association test; FDR: false discovery rate.

**Table S5.** Two-sample Mendelian randomization analysis for proteins showing significant associations identified in our study, excluding HBB (FEV_1_ to proteins).

| Method | No. SNPs | beta | SE | *P* |
| --- | --- | --- | --- | --- |
| Serum levels of protein CCDC80 | | | | |
| IVW | 206 | -0.067 | 0.084 | 0.429 |
| MR-Egger | 206 | 0.145 | 0.274 | 0.597 |
| MR-PRESSO | 206 | -0.067 | 0.084 | 0.430 |
| GSMR | 206 | -0.068 | 0.084 | 0.416 |
| Serum levels of protein IGFBP4 | | | | |
| IVW | 206 | 0.053 | 0.095 | 0.058 |
| MR-Egger | 206 | -0.463 | 0.306 | 0.132 |
| MRPRESSO | 206 | 0.053 | 0.095 | 0.058 |
| GSMR | 206 | 0.046 | 0.091 | 0.615 |

Note: HBB: Hemoglobin subunit beta; FEV_1_: forced expiratory volume in one second; No. SNPs: number of single nucleotide polymorphisms; SE: standard error; IVW: inverse-variance weighted; MR: Mendelian randomization; MR-PRESSO: MR-Pleiotropy Residual Sum and Outlier; GSMR: Generalized Summary-data-based Mendelian Randomization.

**Table S6.** Two-sample Mendelian randomization analysis for proteins showing significant associations identified in our study, excluding HBB (proteins to FEV_1_).

| Method | No. SNPs | beta | SE | *P* |
| --- | --- | --- | --- | --- |
| Serum levels of protein CCDC80 | | | | |
| IVW | 16 | -0.008 | 0.008 | 0.302 |
| MR-Egger | 16 | 0.001 | 0.021 | 0.957 |
| MR-PRESSO | 16 | -0.008 | 0.008 | 0.318 |
| GSMR | 16 | -0.007 | 0.006 | 0.232 |
| Serum levels of protein IGFBP4 | | | | |
| IVW | 6 | -0.010 | 0.010 | 0.369 |
| MR-Egger | 6 | -0.010 | 0.023 | 0.677 |
| MRPRESSO | 6 | -0.009 | 0.010 | 0.410 |
| GSMR | 6 | -0.008 | 0.008 | 0.329 |

Note: HBB: Hemoglobin subunit beta; FEV_1_: forced expiratory volume in one second; No. SNPs: number of single nucleotide polymorphisms; SE: standard error; IVW: inverse-variance weighted; MR: Mendelian randomization; MR-PRESSO: MR-Pleiotropy Residual Sum and Outlier; GSMR: Generalized Summary-data-based Mendelian Randomization.

**Table S7.** Two-sample Mendelian randomization for all immunoglobulin family proteins (FEV_1_ to proteins).

| Method | No. SNPs | beta | SE | *P* |
| --- | --- | --- | --- | --- |
| Serum levels of protein IGHA1; IGHA2; JCHAIN; IGL; IGK; PIGR [1] | | | | |
| IVW | 206 | -0.227 | 0.098 | 0.020 |
| MR-Egger | 206 | -0.349 | 0.319 | 0.275 |
| MR-PRESSO | 206 | -0.227 | 0.098 | 0.021 |
| GSMR | 206 | -0.239 | 0.088 | 0.007 |
| Serum levels of protein IGHA1; IGHA2; JCHAIN; IGL; IGK; PIGR [2] | | | | |
| IVW | 205 | -0.193 | 0.093 | 0.038 |
| MR-Egger | 205 | -0.305 | 0.304 | 0.317 |
| MRPRESSO | 205 | -0.193 | 0.093 | 0.040 |
| GSMR | 205 | -0.230 | 0.087 | 0.008 |
| Serum levels of protein IGHG1; IGHG2; IGHG3; IGHG4; IGL; IGK [1] | | | | |
| IVW | 206 | -0.169 | 0.081 | 0.038 |
| MR-Egger | 206 | -0.231 | 0.264 | 0.382 |
| MRPRESSO | 206 | -0.169 | 0.081 | 0.038 |
| GSMR | 206 | -0.153 | 0.085 | 0.070 |
| Serum levels of protein IGHG1; IGHG2; IGHG3; IGHG4; IGL; IGK [2] | | | | |
| IVW | 206 | -0.135 | 0.088 | 0.125 |
| MR-Egger | 206 | -0.430 | 0.285 | 0.134 |
| MRPRESSO | 206 | -0.134 | 0.088 | 0.127 |
| GSMR | 206 | -0.135 | 0.089 | 0.129 |
| Serum levels of protein IGHG4 | | | | |
| IVW | 206 | 0.120 | 0.089 | 0.178 |
| MR-Egger | 206 | 0.383 | 0.290 | 0.188 |
| MRPRESSO | 206 | 0.120 | 0.089 | 0.180 |
| GSMR | 206 | 0.097 | 0.089 | 0.277 |
| Serum levels of protein IGHM; JCHAIN; IGL; IGK | | | | |
| IVW | 206 | -0.054 | 0.095 | 0.566 |
| MR-Egger | 206 | -0.047 | 0.309 | 0.877 |
| MRPRESSO | 206 | -0.054 | 0.095 | 0.566 |
| GSMR | 206 | -0.051 | 0.092 | 0.583 |
| Serum levels of protein IGHG2 | | | | |
| IVW | 206 | 0.054 | 0.094 | 0.567 |
| MR-Egger | 206 | -0.041 | 0.305 | 0.894 |
| MRPRESSO | 206 | 0.054 | 0.094 | 0.567 |
| GSMR | 206 | 0.022 | 0.090 | 0.810 |
| Serum levels of protein IGHE; IGL; IGK | | | | |
| IVW | 205 | -0.002 | 0.100 | 0.985 |
| MR-Egger | 205 | 0.018 | 0.327 | 0.956 |
| MRPRESSO | 205 | -0.002 | 0.100 | 0.985 |
| GSMR | 205 | -0.031 | 0.093 | 0.735 |
| Serum levels of protein IGHD; IGL; IGK | | | | |
| IVW | 205 | 0.011 | 0.094 | 0.910 |
| MR-Egger | 205 | 0.490 | 0.308 | 0.113 |
| MRPRESSO | 205 | 0.011 | 0.094 | 0.910 |
| GSMR | 205 | -0.027 | 0.094 | 0.778 |

Note: FEV_1_: forced expiratory volume in one second; No. SNPs: number of single nucleotide polymorphisms; SE: standard error; IVW: inverse-variance weighted; MR: Mendelian randomization; MR-PRESSO: MR-Pleiotropy Residual Sum and Outlier; GSMR: Generalized Summary-data-based Mendelian Randomization.

**Table S8.** Two-sample Mendelian randomization for all immunoglobulin family proteins (proteins to FEV_1_).

| Method | No. SNPs | beta | SE | *P* |
| --- | --- | --- | --- | --- |
| Serum levels of protein IGHA1; IGHA2; JCHAIN; IGL; IGK; PIGR [1] | | | | |
| IVW | 22 | -0.012 | 0.006 | 0.028 |
| MR-Egger | 22 | -0.017 | 0.012 | 0.192 |
| MR-PRESSO | 22 | -0.012 | 0.006 | 0.039 |
| GSMR | 22 | -0.010 | 0.005 | 0.031 |
| Serum levels of protein IGHA1; IGHA2; JCHAIN; IGL; IGK; PIGR [2] | | | | |
| IVW | 25 | -0.002 | 0.006 | 0.638 |
| MR-Egger | 25 | 0.016 | 0.019 | 0.412 |
| MRPRESSO | 25 | -0.003 | 0.006 | 0.642 |
| GSMR | 25 | -0.001 | 0.005 | 0.804 |
| Serum levels of protein IGHG1; IGHG2; IGHG3; IGHG4; IGL; IGK [1] | | | | |
| IVW | 9 | 0.009 | 0.010 | 0.379 |
| MR-Egger | 9 | -0.025 | 0.028 | 0.403 |
| MRPRESSO | 9 | 0.009 | 0.010 | 0.405 |
| GSMR | 9 | 0.008 | 0.008 | 0.321 |
| Serum levels of protein IGHG1; IGHG2; IGHG3; IGHG4; IGL; IGK [2] | | | | |
| IVW | 15 | 0.006 | 0.006 | 0.291 |
| MR-Egger | 15 | -0.019 | 0.015 | 0.250 |
| MRPRESSO | 15 | 0.006 | 0.006 | 0.293 |
| GSMR | 15 | 0.006 | 0.006 | 0.315 |
| Serum levels of protein IGHG4 | | | | |
| IVW | 6 | -0.001 | 0.012 | 0.964 |
| MR-Egger | 6 | 0.060 | 0.031 | 0.123 |
| MRPRESSO | 6 | -0.001 | 0.012 | 0.966 |
| GSMR | 6 | -0.0003 | 0.009 | 0.967 |
| Serum levels of protein IGHM; JCHAIN; IGL; IGK | | | | |
| IVW | 30 | -0.0002 | 0.005 | 0.973 |
| MR-Egger | 30 | -0.002 | 0.013 | 0.852 |
| MRPRESSO | 30 | -0.0002 | 0.005 | 0.973 |
| GSMR | 30 | -0.001 | 0.004 | 0.886 |
| Serum levels of protein IGHG2 | | | | |
| IVW | 6 | 0.007 | 0.012 | 0.535 |
| MR-Egger | 6 | 0.034 | 0.021 | 0.185 |
| MRPRESSO | 6 | 0.007 | 0.012 | 0.562 |
| GSMR | 6 | 0.007 | 0.008 | 0.391 |
| Serum levels of protein IGHE; IGL; IGK | | | | |
| IVW | 18 | 0.012 | 0.005 | 0.021 |
| MR-Egger | 18 | 0.016 | 0.016 | 0.364 |
| MRPRESSO | 18 | 0.012 | 0.005 | 0.034 |
| GSMR | 18 | 0.013 | 0.005 | 0.008 |
| Serum levels of protein IGHD; IGL; IGK | | | | |
| IVW | 14 | -0.012 | 0.008 | 0.135 |
| MR-Egger | 14 | 0.011 | 0.021 | 0.608 |
| MRPRESSO | 14 | -0.012 | 0.008 | 0.159 |
| GSMR | 14 | -0.010 | 0.006 | 0.097 |

Note: FEV_1_: forced expiratory volume in one second; No. SNPs: number of single nucleotide polymorphisms; SE: standard error; IVW: inverse-variance weighted; MR: Mendelian randomization; MR-PRESSO: MR-Pleiotropy Residual Sum and Outlier; GSMR: Generalized Summary-data-based Mendelian Randomization.

## **Supplemental Figures**

**Figure S1.** Volcano plot for associations between decline rate of FEV_1_ and proteins.


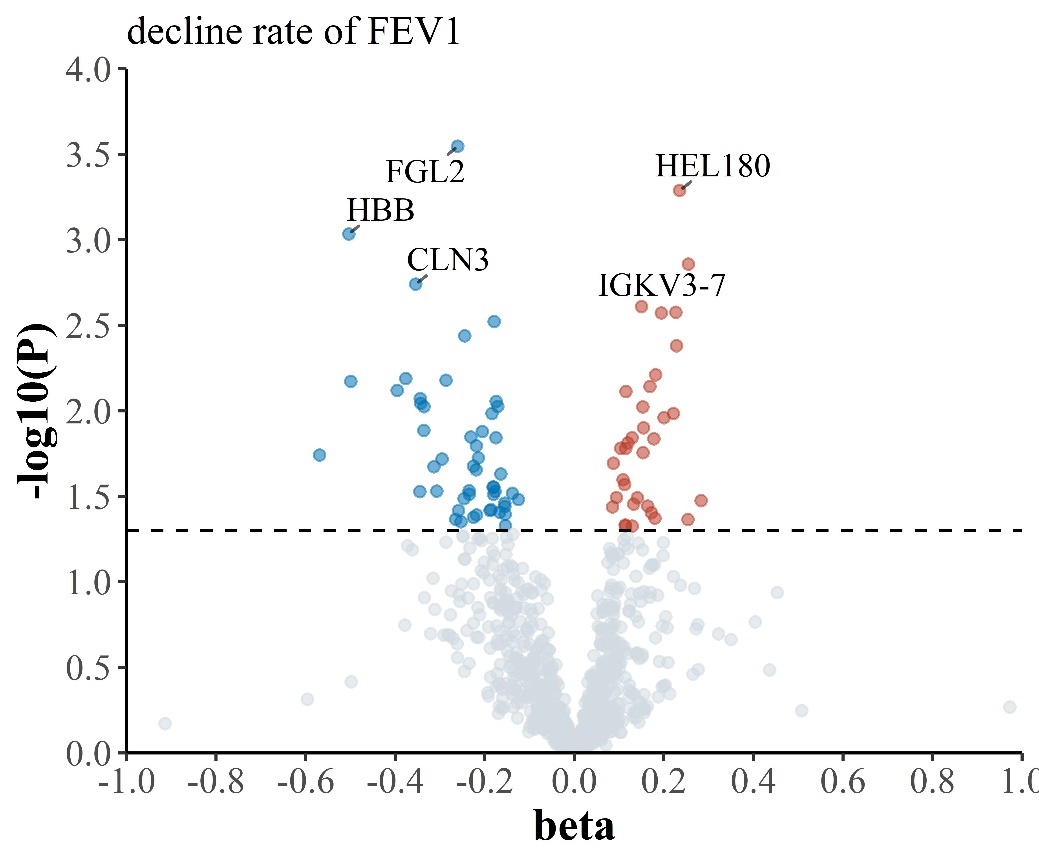


Note: FEV_1_: forced expiratory volume in one second.

**Figure S2.** Elbow plot for K-means clustering.


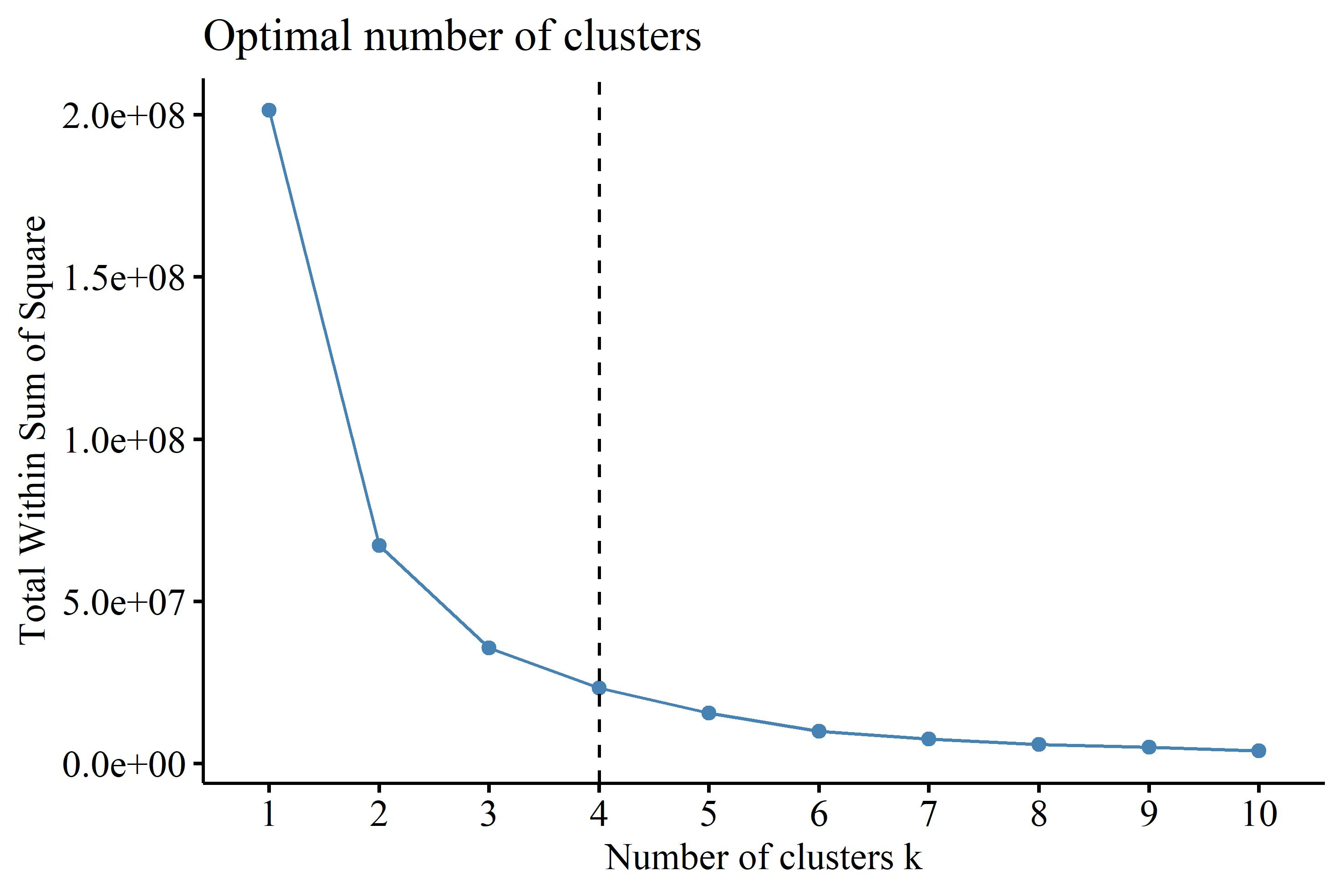


**Figure S3.** Average linear trajectories of FEV_1_ in each cluster.


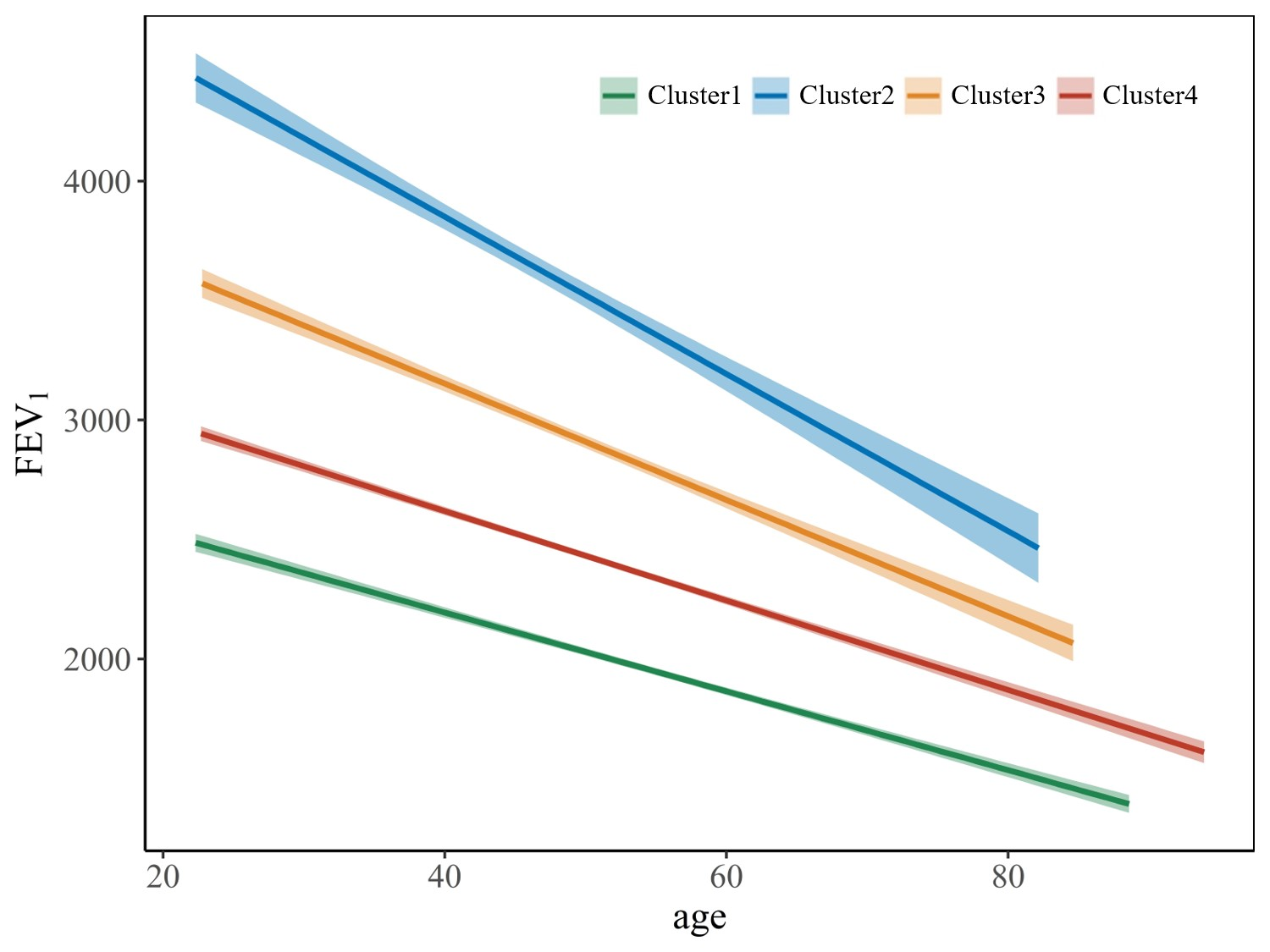


Note: FEV_1_: forced expiratory volume in one second.

**Figure S4.** Volcano plots of cluster-based model (cluster2 as reference cluster), showing the gene name of top 5 significant proteins, where dashed line represents the nominal significant level (*P* < 0.05).


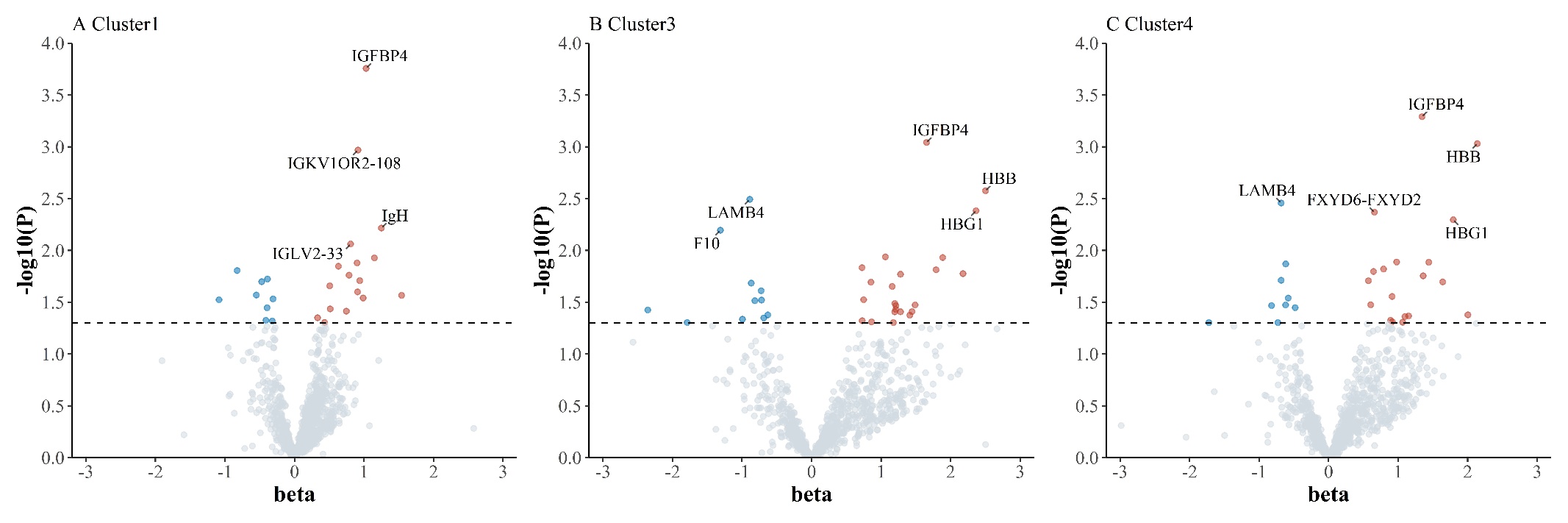


**A** Effect of cluster1 compared to cluster2 on proteins; **B** Effect of cluster3 compared to cluster2 on proteins; **C** Effect of cluster4 compared to cluster2 on proteins.

**Figure S5.** Volcano plots of restricted cubic spline model, showing the gene name of top 5 significant proteins, where dashed line represents the nominal significant level (*P* < 0.05).


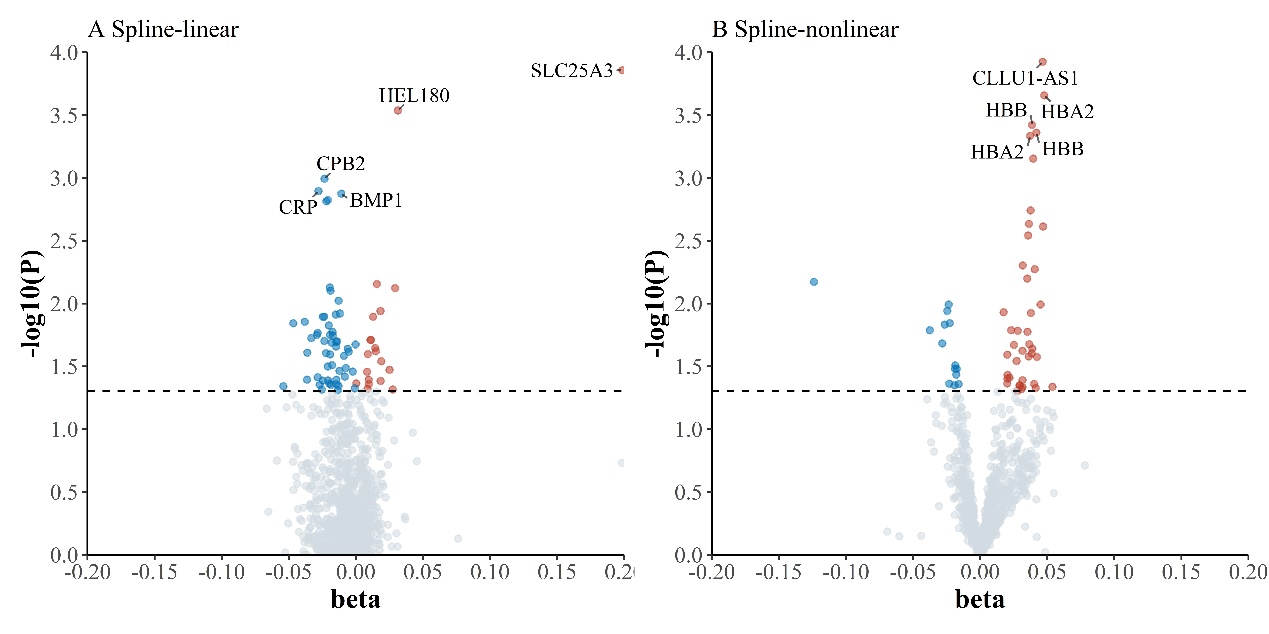


**A** linear effects of the slopes of FEV_1_ trajectories; **B** nonlinear effects of the slope of FEV_1_ trajectories. Note: FEV_1_: forced expiratory volume in one second.

**Figure S6.** Average and individual (gray) trajectories of FEV_1_ in each latent class using latent class mixed model.


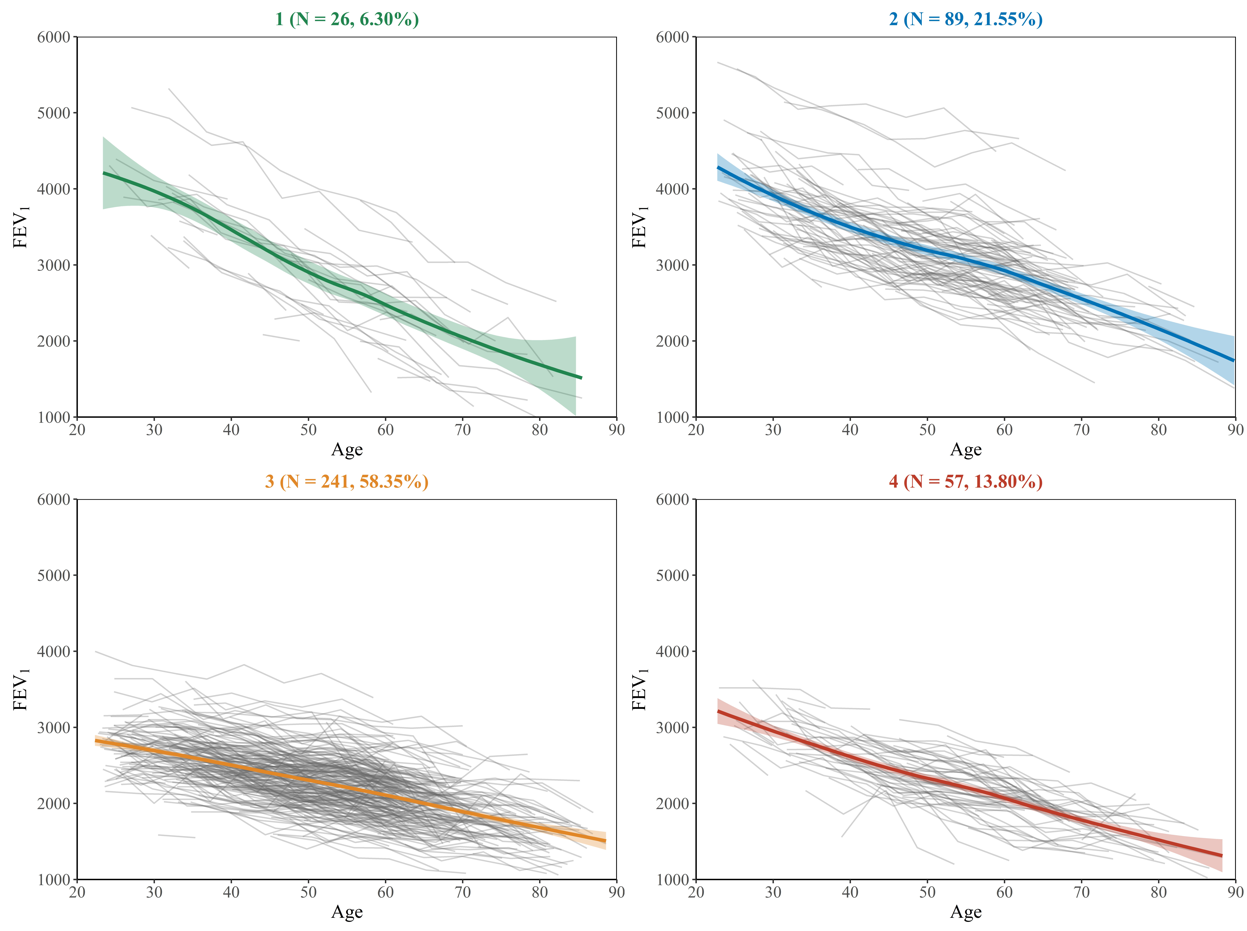


Note: FEV_1_: forced expiratory volume in one second.

**Figure S7.** Summary of average trajectories of FEV_1­_ in each latent class using latent class mixed model.


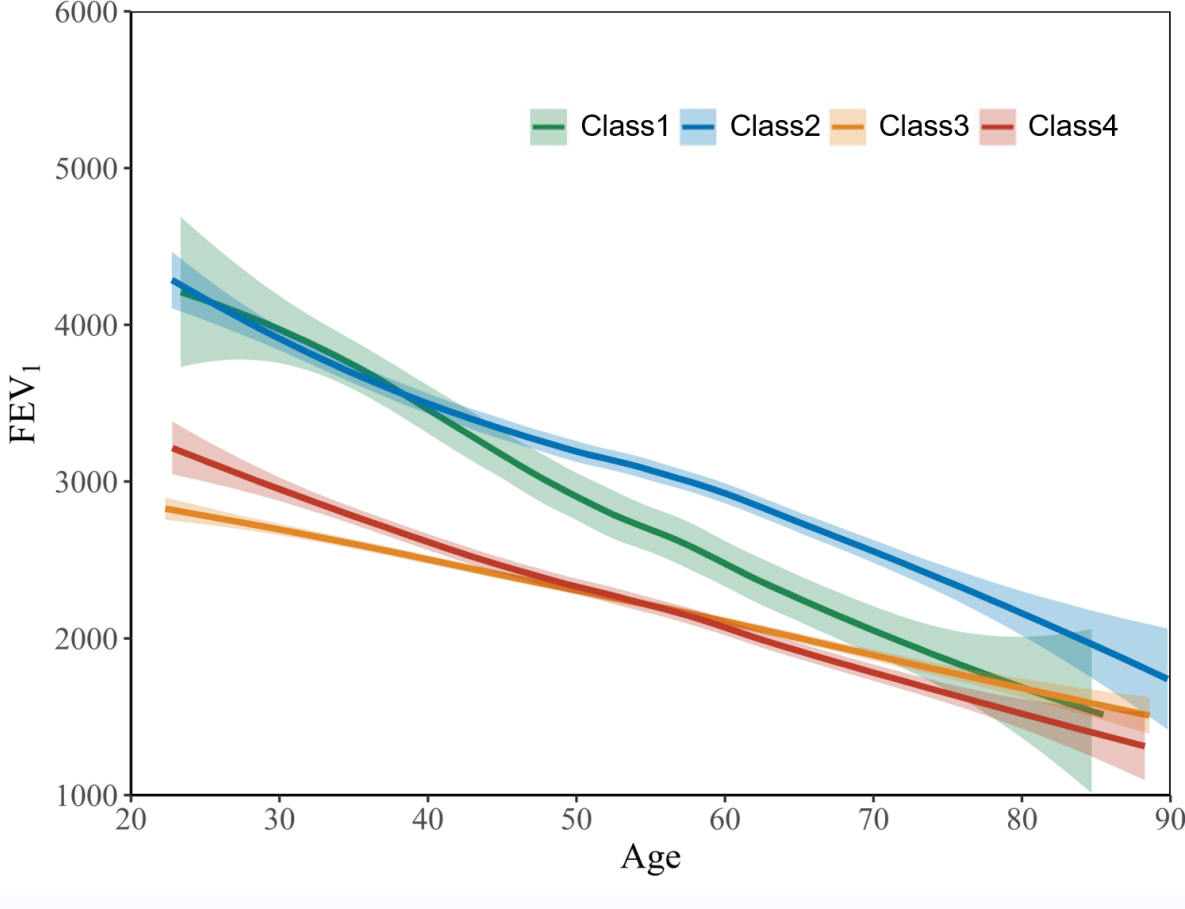


Note: FEV_1_: forced expiratory volume in one second.

**Figure S8.** Volcano plots of latent class mixed model (class1 as reference cluster), showing the gene name of top 5 significant proteins, where dashed line represents the nominal significant level (*P* < 0.05).


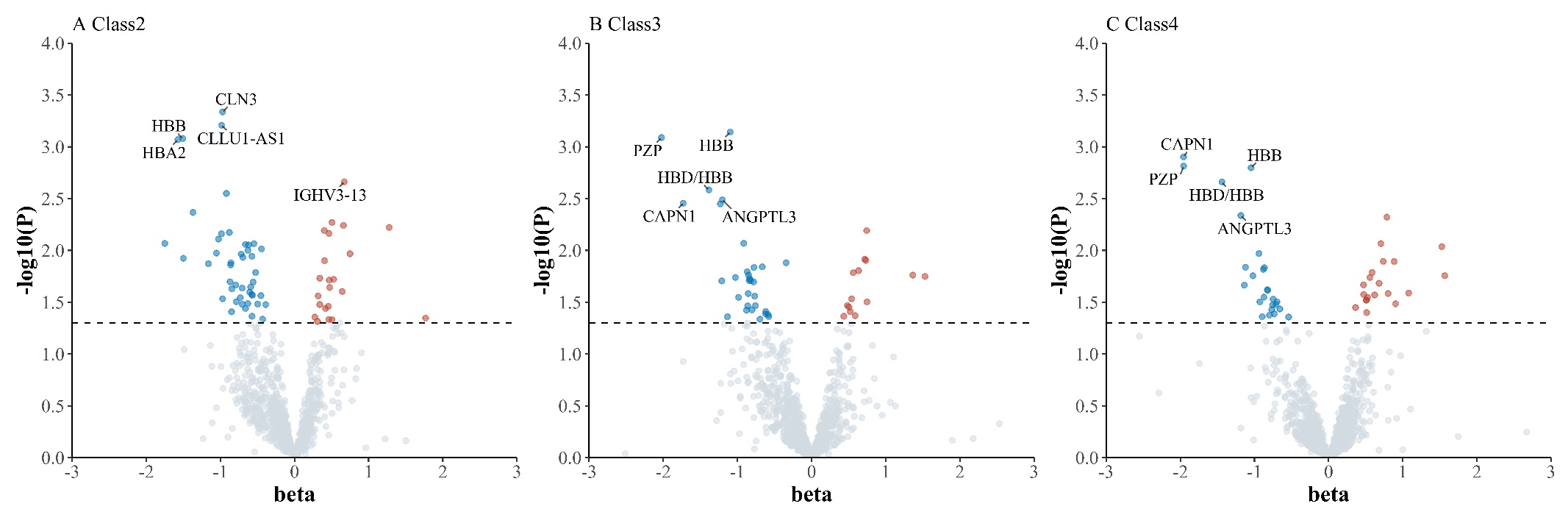


**A** Effect of class2 compared to cluster1 on proteins; **B** Effect of class3 compared to class1 on proteins; **C** Effect of class4 compared to class1 on proteins.

**Figure S9.** Volcano plots of mixed model for repeated measurements, with protein effects at specific ages, showing the name of top 5 significant proteins, where two dashed lines represent the nominal significant level (*P* < 0.05) and the FDR-corrected significant level (FDR-*q* < 0.05), respectively.


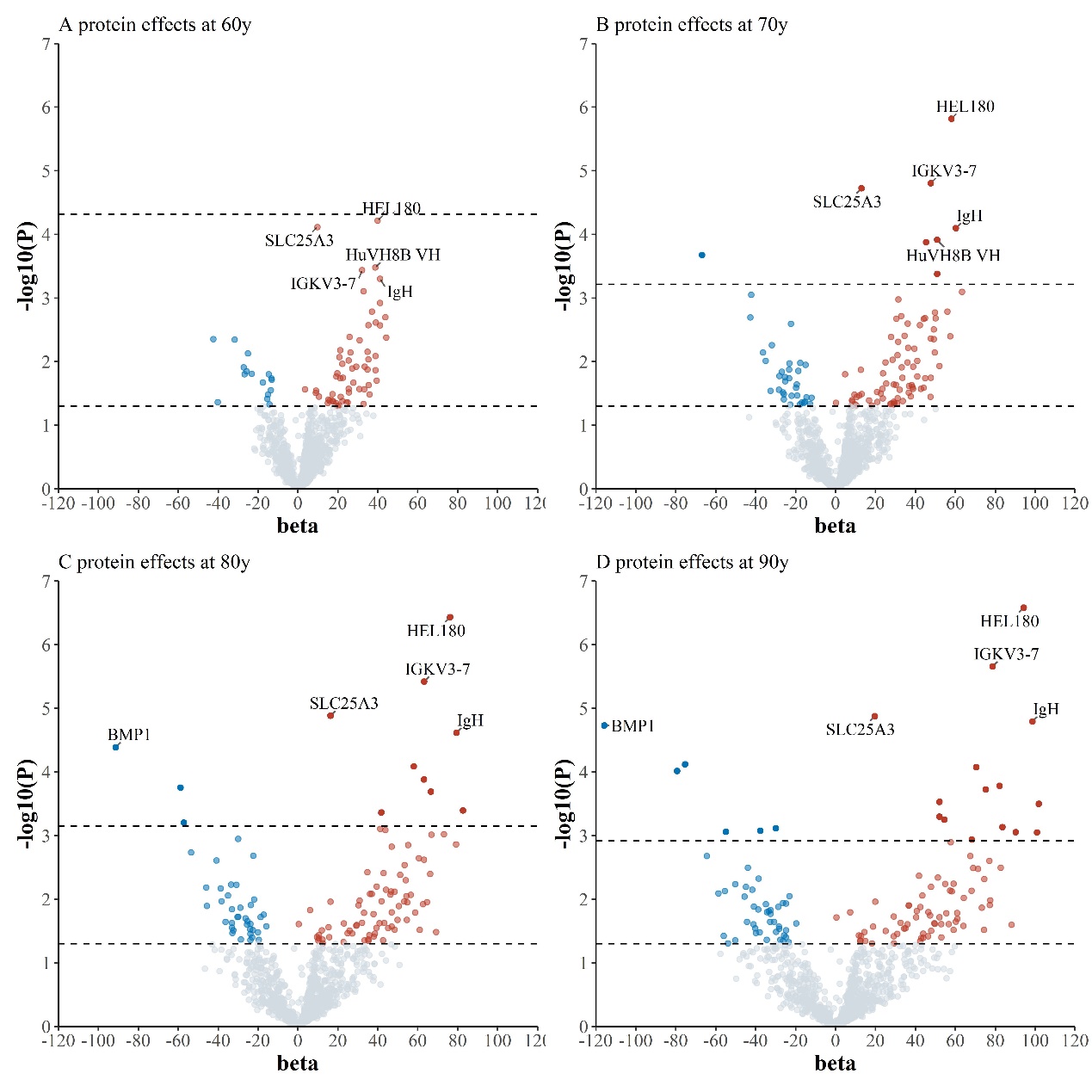


**A** Effect of proteins on FEV_1_ at 60 years old; **B** Effect of proteins on FEV_1_ at 70 years old; **C** Effect of proteins on FEV_1_ at 80 years old; **D** Effect of proteins on FEV_1_ at 90 years old. Note: FEV_1_: forced expiratory volume in one second; FDR: false discovery rate.

**Figure S10.** Volcano plot of mixed model for repeated measurements, with the effects of protein*age interactions, showing the name of top 5 significant proteins, where two dashed lines represent the nominal significant level (*P* < 0.05) and the FDR-corrected significant level (FDR-*q* < 0.05), respectively.


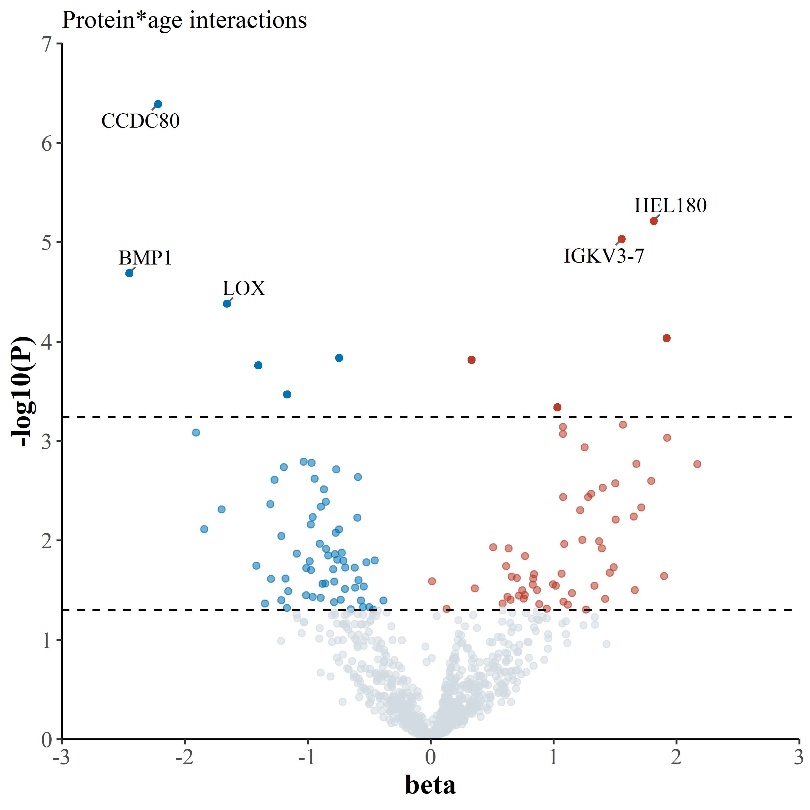


Note: FDR: false discovery rate.

**Figure S11.** Volcano plot of proteomic from UK Biobank in exploratory study, showing the name of top 5 significant proteins, where two dashed lines represent the nominal significant level (*P* < 0.05) and the FDR-corrected significant level (FDR-*q* < 0.05), respectively.


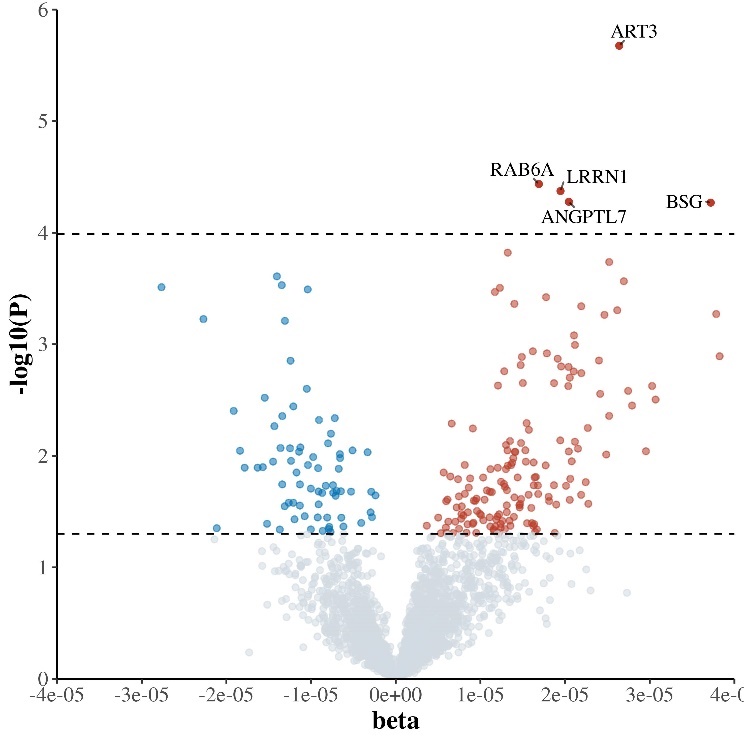


Note: FDR: false discovery rate.

**Figure S12.** Scatter plot of two-sample Mendelian randomization (HBB to FEV_1_).


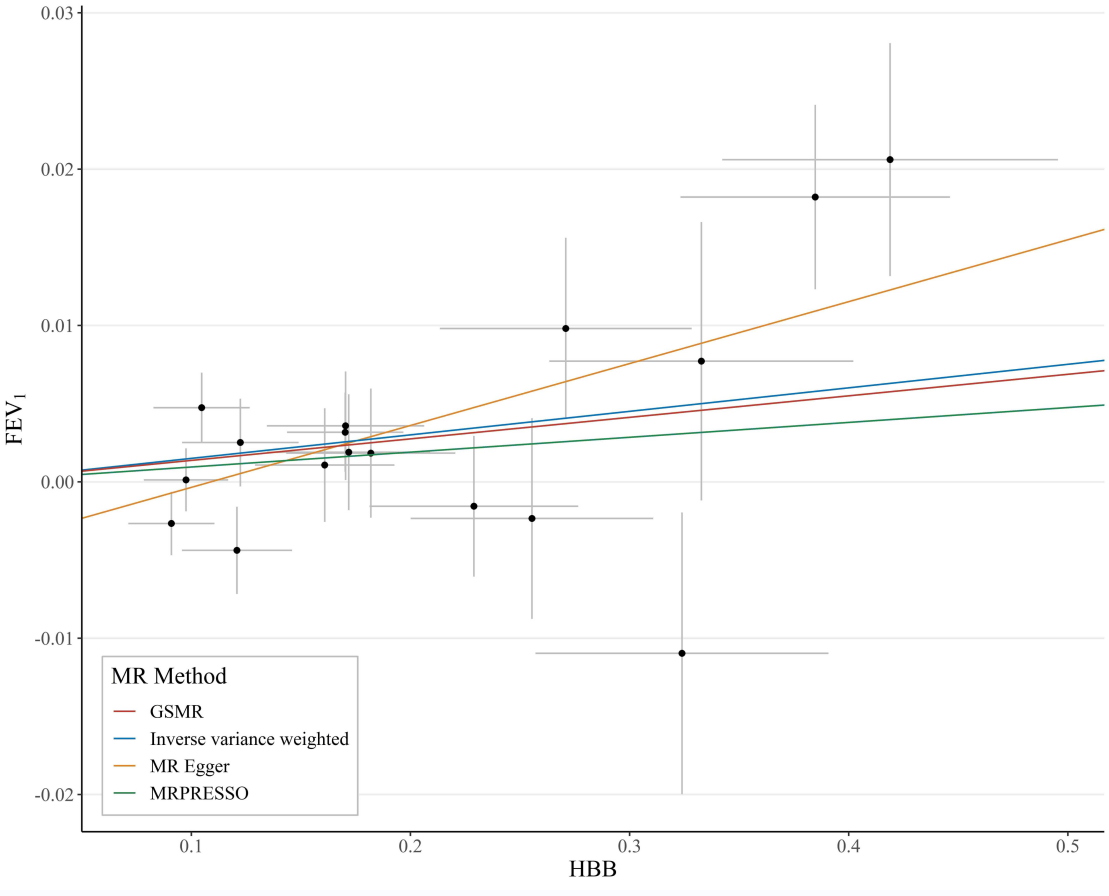


Notes: HBB: Hemoglobin subunit beta; FEV_1_: forced expiratory volume in one second; MR: Mendelian randomization; GSMR: Generalized Summary-data-based Mendelian Randomization; MR-PRESSO: MR-Pleiotropy Residual Sum and Outlier.

**Figure S13.** Scatter plot of two-sample Mendelian randomization (FEV_1_ to HBB).


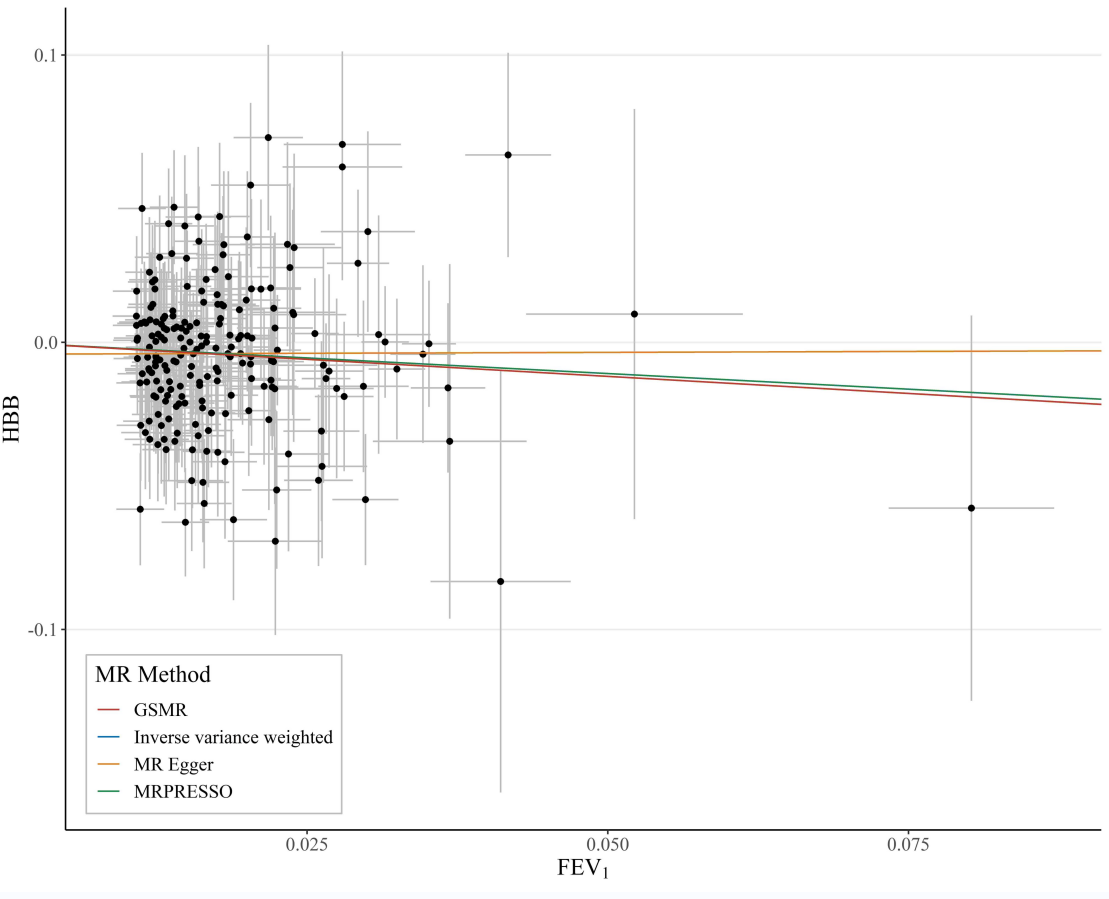


Notes: HBB: Hemoglobin subunit beta; FEV_1_: forced expiratory volume in one second; MR: Mendelian randomization; GSMR: Generalized Summary-data-based Mendelian Randomization; MR-PRESSO: MR-Pleiotropy Residual Sum and Outlier.
